# 2D Raman, ATR-FTIR, WAXD, SAXS and DSC data of PET mono- and PET/PA6 bicomponent filaments

**DOI:** 10.1016/j.dib.2021.107416

**Published:** 2021-09-23

**Authors:** K. Sharma, O. Braun, S. Tritsch, R. Muff, R. Hufenus, E. Perret

**Affiliations:** a Laboratory for Advanced Fibers, Empa, Swiss Federal Laboratories for Materials Science and Technology, Lerchenfeldstrasse 5, St. Gallen 9014, Switzerland; b KTH Royal Institute of Technology, Stockholm 114 16 Sweden; c Transport at Nanoscale Interfaces Laboratory, Empa, Swiss Federal Laboratories for Materials Science and Technology, Überlandstrasse 129, Dübendorf 8600, Switzerland; d Department of Physics, University of Basel, Klingelbergstrasse 82, Basel 4056, Switzerland; e Hochschule Reutlingen, Alteburgstrasse 150, Reutlingen 72762, Germany; f Center for X-ray Analytics, Empa, Swiss Federal Laboratories for Materials Science and Technology, Überlandstrasse 129, Dübendorf 8600, Switzerland

**Keywords:** Raman mapping, PET fibers, X-ray diffraction, Infrared spectroscopy, Crystallinity, Radial gradients

## Abstract

This data in brief article summarizes structural data obtained from monocomponent melt-spun and offline drawn poly(ethylene terephthalate) (PET) monofilaments, as well as from melt-spun bicomponent core-sheath PET-polyamide 6 (PA6) filaments. The diameters of the single filaments range from 27 µm to 79 µm. Presented analysis techniques and results thereof are (i) Raman mapping of filament cross-sections: 2D maps of peak positions, widths, peak area ratios; (ii) attenuated total reflection Fourier transform infrared spectroscopy (ATR-FTIR): ATR-FTIR spectra and extraction of surface crystallinity; (iii) wide-angle x-ray diffraction (WAXD): WAXD patterns and extraction of average crystallinity; (iv) small-angle x-ray scattering (SAXS): SAXS patterns and determined crystallite sizes and long-spacings; (v) differential scanning calorimetry (DSC): thermograms and extracted average crystallinity as well as thermal properties; (vi) atomic force microscopy (AFM): AFM image of the surface of an embedded fiber cross-section. For more information, see the publication by E. Perret et al. 'High-resolution 2D Raman mapping of mono- and bicomponent filament cross-sections' [1].

## Specifications Table

**Table:** 

Subject	Materials Science: Polymers and Plastics
Specific subject area	Structure of melt-spun mono- and bicomponent filaments.
Type of data	Table Image Figure Equations
How data were acquired	Instruments: Raman data: WITec Alpha 300 R confocal Raman microscope (WITec GmbH, Ulm, Germany) in backscattering geometry. ATR-FTIR data: Bruker Tensor 27 (Bruker Optics, Ettlingen, Germany) with a single Reflection attenuated total reflectance (GladiATR™) accessory (Pike Technologies, Fitchburg, Wisconsin, United States) X-ray data (WAXD/SAXS): Bruker Nanostar U diffractometer (Bruker AXS, Karlsruhe, Germany) DSC data: DSC 214 Polyma (Netzsch, Selb, Germany) AFM data: Nanoscope (Bruker AXS, Karlsruhe Germany) Software: Raman analyis: Python codes, WITec Project (Version 5.1, WITec GmbH, Ulm, Germany) ATR-FTIR analysis: OPUS™ software (Version 8.5, Bruker AXS, Karlsruhe, Germany), Python codes X-ray data analysis: DIFFRAC.EVA (version 4.2., Bruker AXS, Karlsruhe, Germany), Python codes DSC analysis: NETZSCH Proteus Thermal Analysis software (Version 7.1.0, Selb, Germany) AFM analysis: AFM NanoScope Analysis (Version 1.9, Bruker AXS, Karlsruhe, Germany)
Data format	Raw Analyzed
Parameters for data collection	2D Raman maps have been acquired of filament cross-sections using a blue laser (λ = 488 nm) and a WITec Alpha 300 R confocal Raman microscope (WITec GmbH, Ulm, Germany). ATR-FTIR data was collected from differently drawn PET monofilaments with a Bruker Tensor 27 FTIR spectrometer. For each fiber, 32 scans have been averaged and the spectral resolution was 2 cm^−1^. WAXD and SAXS patterns of all filaments were recorded on a Bruker Nanostar U diffractometer (Bruker AXS, Karlsruhe, Germany) with Cu-Kα radiation (λ = 1.5419 Å) and a VÅNTEC-2000 MikroGap area detector. The beam defining pinhole was 300 µm. Thermal properties were measured for all filaments with the DSC 214 Polyma (Netzsch, Selb, Germany) in a nitrogen atmosphere (40 mL/min) and a ramping rate of 10 °C/min. A 2D AFM map was acquired of one filament cross-section using a scanning speed of 0.5 Hz (512 × 512 points) and the AFM Nanoscope (Bruker AXS, Karlsruhe Germany).
Description of data collection	Fibers have been embedded in a resin hardener followed by a polishing of the samples. The polished cross-sections have subsequently been analyzed with Raman mapping by scanning the samples through *a* < 1 µm sized laser beam. Special attention was given to variations in Raman peak heights, areas, positions and widths throughout the fiber cross-sections. ATR-FTIR spectra of as-spun and offline drawn PET filaments have been collected with the Bruker Tensor 27 (Bruker Optics, Ettlingen, Germany) using a single Reflection attenuated total reflectance (GladiATR™) accessory. Surface crystallinity values of the PET filaments have been calculated. WAXD and SAXS patterns have been acquired from all filaments and average crystallinity values, as well as long-spacings and crystallite sizes were determined. For the bicomponent filaments, patterns were acquired for the filament with and without the filament's sheath material. Azimuthal and radial profiles of WAXD patterns were analyzed in order to determine the average crystallinities. Transversal and meridional scans were extracted from SAXS patterns and fitted in order to obtain long-spacings and crystallite sizes. Thermal properties of the filaments were analyzed with DSC, by cutting the fibers into small pieces. The crystallinities were calculated from the heat of fusion. AFM was performed on one embedded filament in order to verify the curvature of the sample.
Data source location	Empa, St. Gallen, Switzerland
Data accessibility	Mendeley Data [15] http://dx.doi.org/10.17632/gx9mbxvnf2.2
Related research article	E. Perret, O. Braun, K. Sharma, S. Tritsch, R. Muff, R. Hufenus, High-resolution 2D Raman mapping of mono- and bicomponent filament cross-sections, Polymer, 229 (2021) 124011. http://dx.doi.org/10.1016/10.1016/j.polymer.2021.124011

## Value of the Data

• The presented procedures to extract structural information with different techniques from thin PET filaments are of potential interest to the polymer fiber community.
• The presented embedding procedure of filaments is of potential interest to other researchers for cross-sectional analysis techniques.
• The presented 2D Raman mapping results highlight the power of this technique to obtain information about the 2D microstructure of fiber cross-sections.
• Extraction of surface crystallinity of PET filaments from ATR-FTIR spectra is of potential interest to other researchers.
• The presented x-ray data is useful for the further development of PET fibers.

## 1 Data Description

### 1.1 Raman data

#### 1.1.1 2D maps

**Fig. 1:**
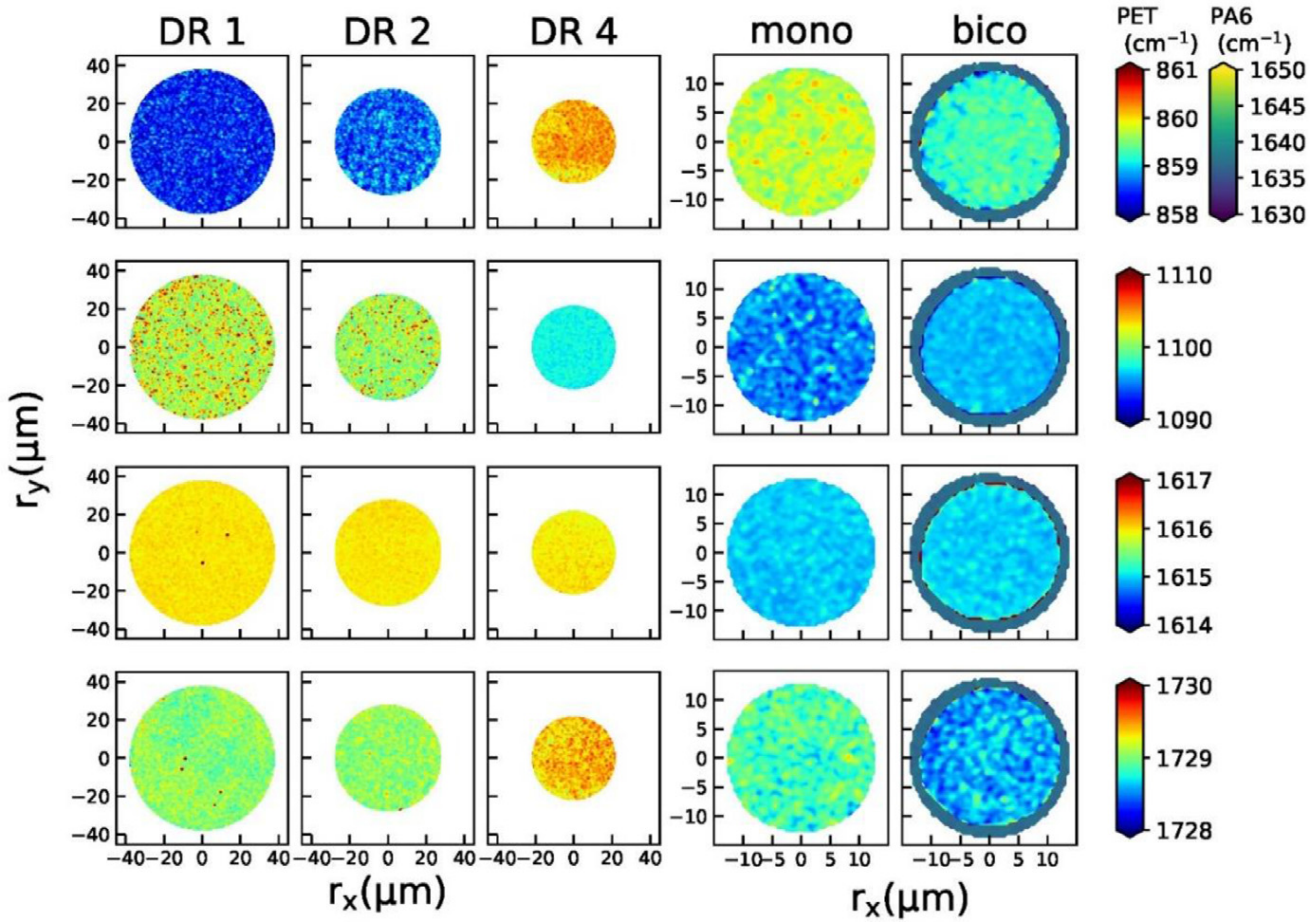
Raman maps of peak positions for all fibers. First row: 860 cm^−1^, second row: 1097 cm^−1^ third row: 1616 cm^−1^, fourth row: 1729 cm^−1^. For the bicomponent filament, the PA6 sheath is illustrated with the position of the 1635 cm^−1^ peak.

**Fig. 2:**
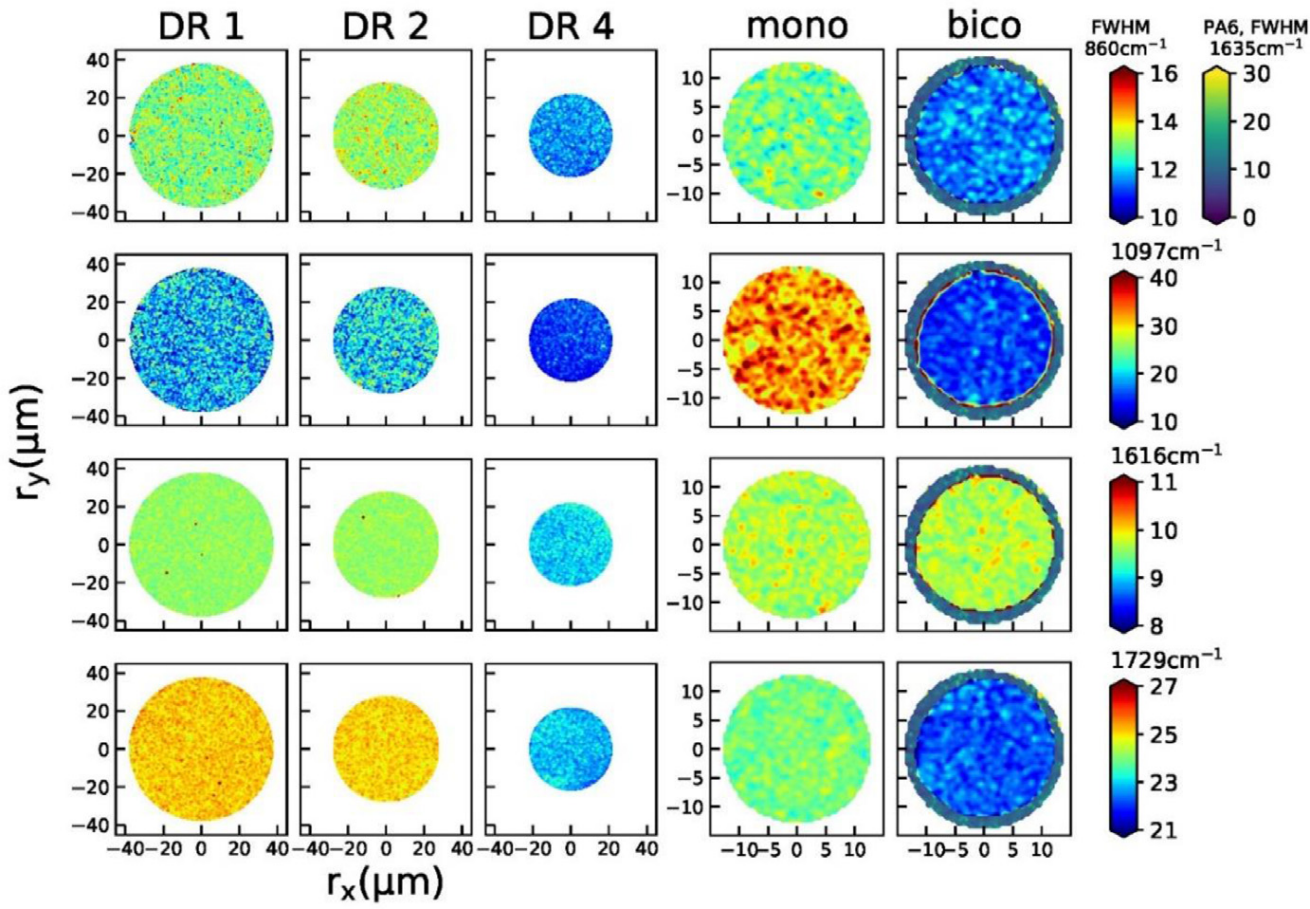
Raman maps of peak widths (FWHM) for all fibers. First row: 860 cm^−1^, second row: 1097 cm^−1^ third row: 1616 cm^−1^, fourth row: 1729 cm^−1^.

**Fig. 3:**
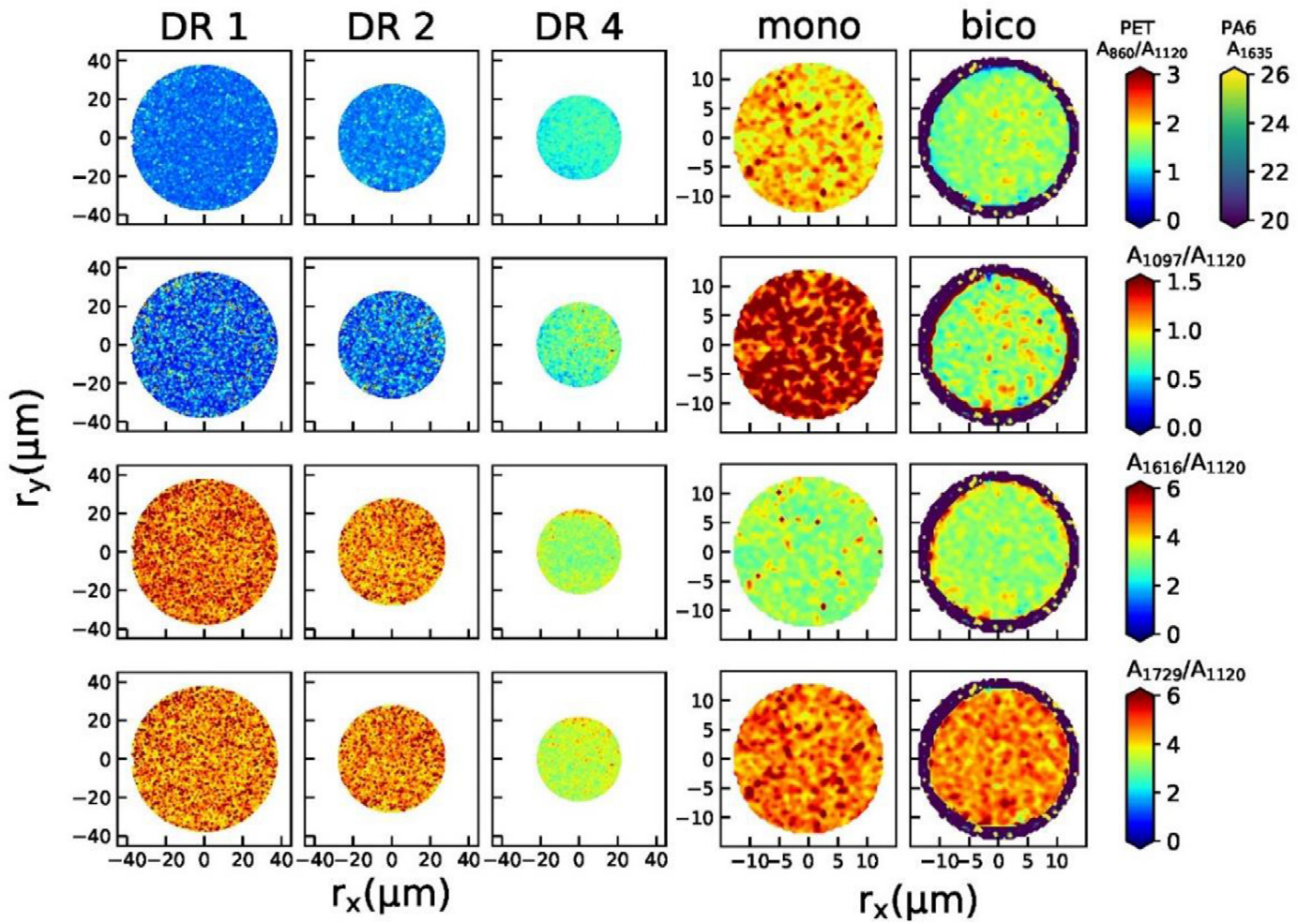
Raman maps of peak area ratios for all fibers.

Fig. 1 shows the Raman maps for all fibers for the 860, 1097, 1616 and 1729 cm^−1^ peak positions. Fig. 2 shows the corresponding peak widths and Fig. 3 the peak area ratios with respect to the 1120 cm^−1^ peak. Note that the maps for fibers DR1, DR2 and DR4 (left panel) span an area of 90 × 90 µm and the ones of the mono and bico fibers span a smaller area of 30 × 30 µm (right panel). For the bico fiber, the PA6 sheath was mapped by analyzing the FWHM of the 1635 cm^−1^ peak. The assignments of the Raman peaks to vibrations in PET are given in the article by Perret et al. [1]. All Raman maps can be found online in the Mendeley repository [15] as text files.

**Fig. 4:**
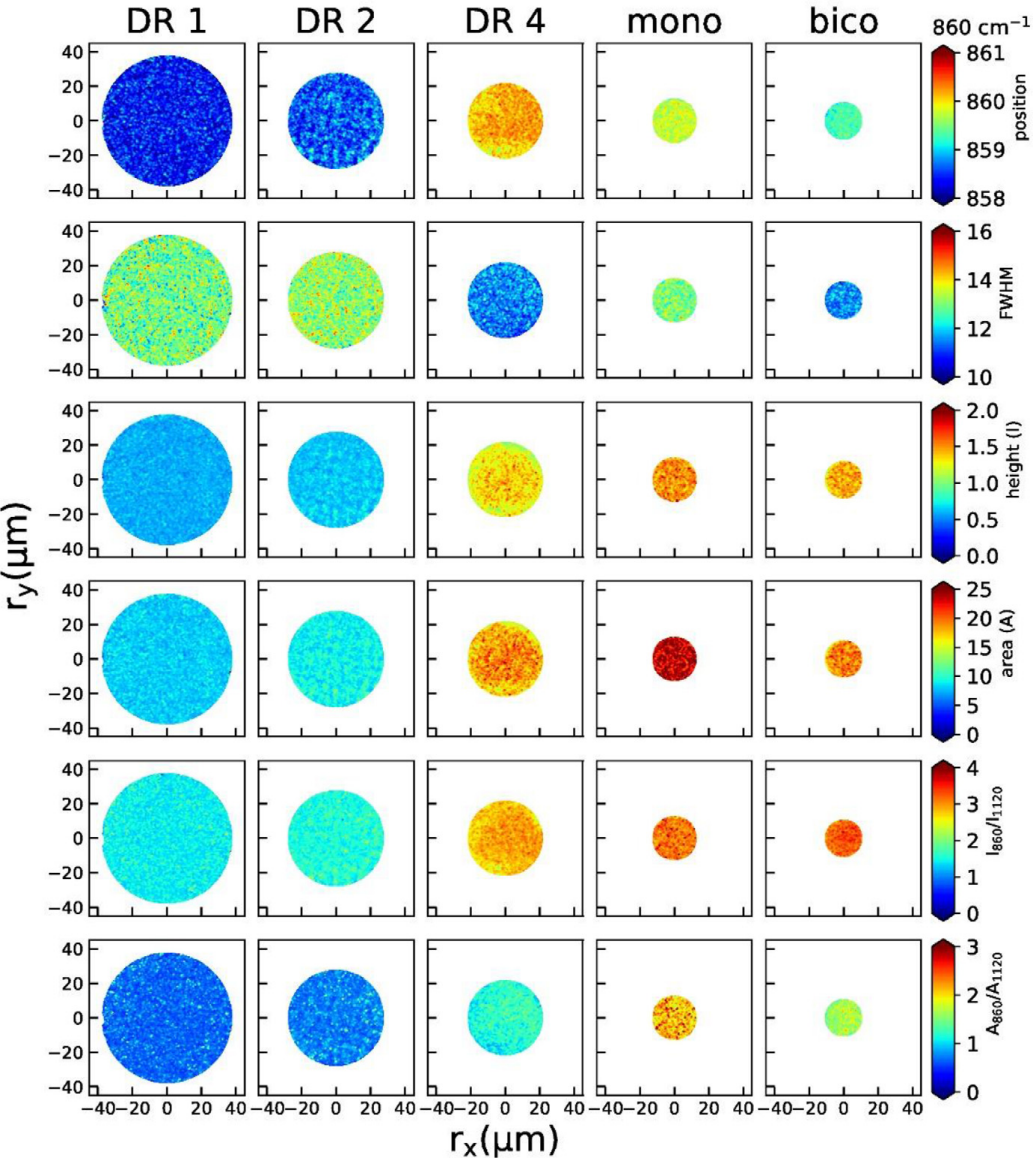
Raman maps from the peak at 860 cm^−1^ (position, width, height, area, peak height ratios and area ratios) for all fibers.

**Fig. 5:**
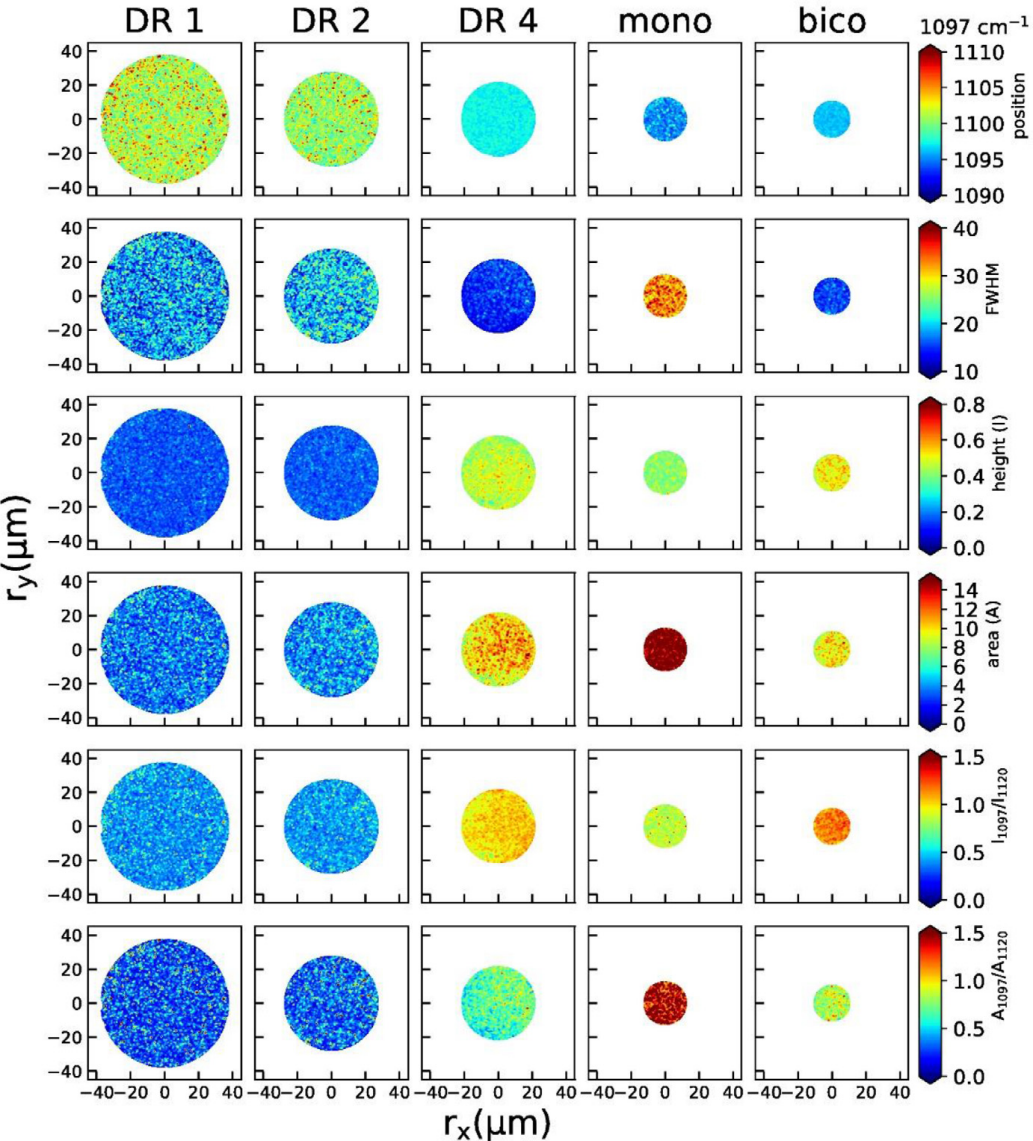
Raman maps from the peak at 1097 cm^−1^ (position, width, height, area, peak height ratios and area ratios) for all fibers.

**Fig. 6:**
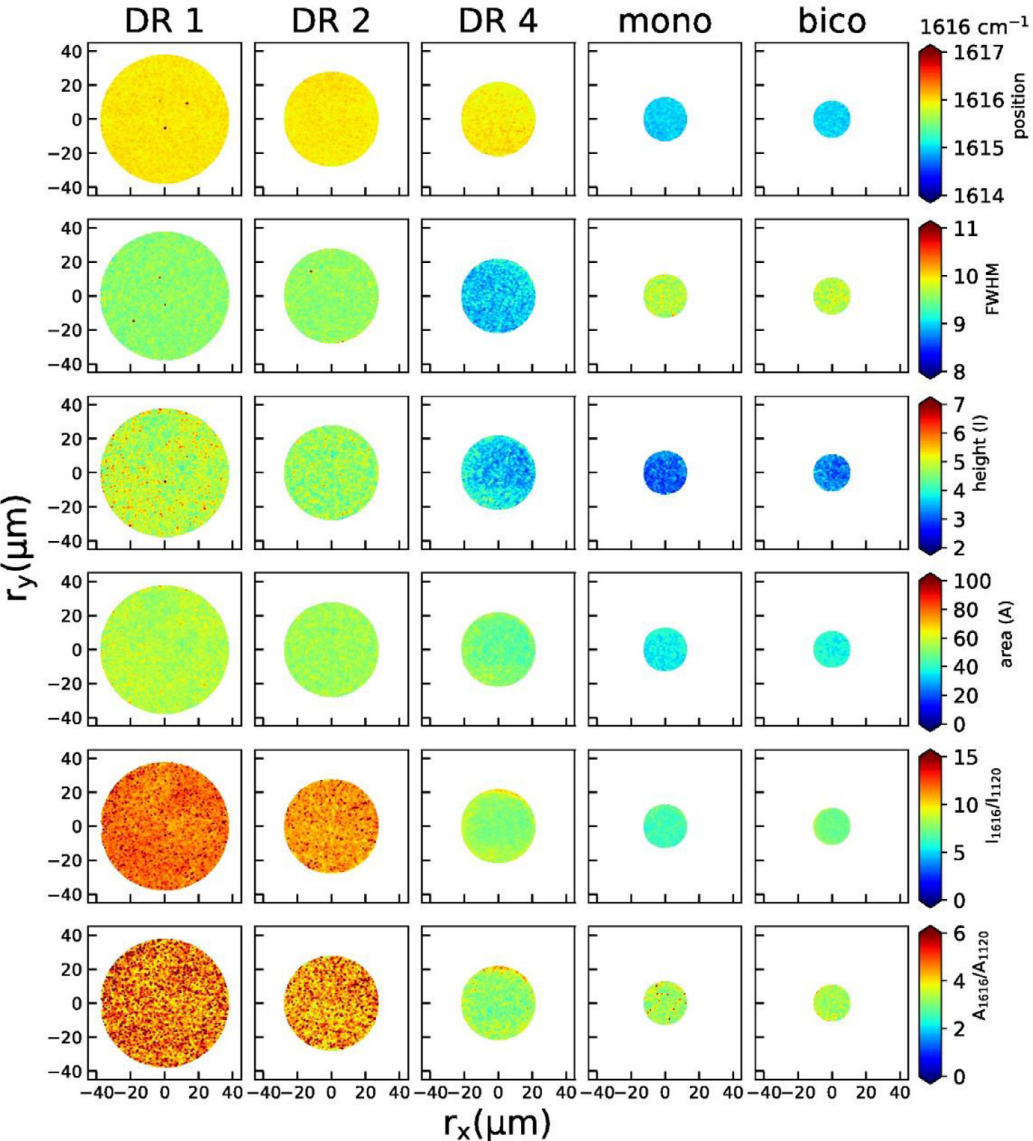
Raman maps from the peak at 1616 cm^−1^ (position, width, height, area, peak height ratios and area ratios) for all fibers.

**Fig. 7:**
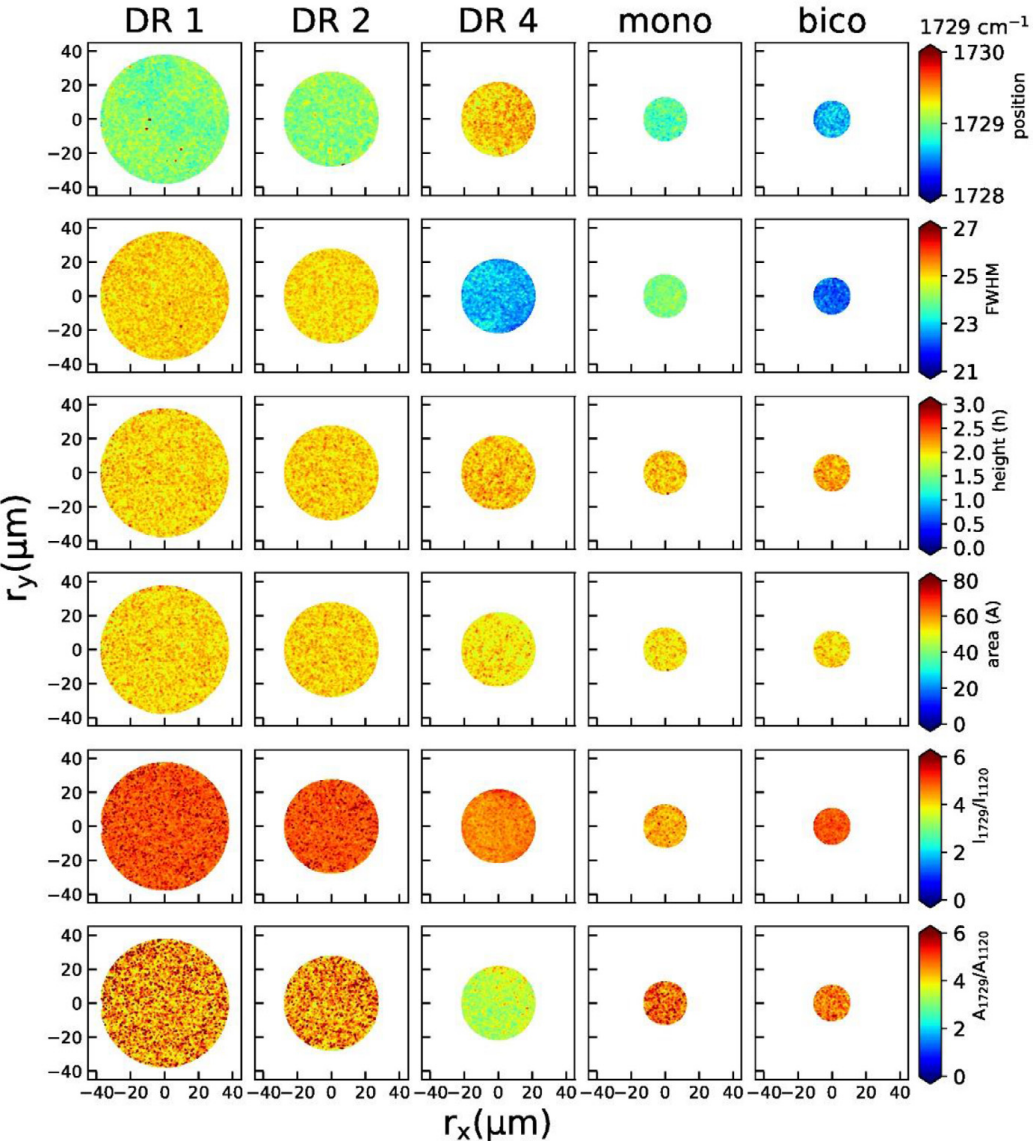
Raman maps from the peak at 1729 cm^−1^ (position, width, height, area, peak height ratios and area ratios) for all fibers.

2D maps of peak positions, widths, heights, areas, height ratios and area ratios of individual peaks are shown below for the 860 cm^−1^ peak (Fig. 4), the 1097 cm^−1^ peak (Fig. 5), the 1616 cm^−1^ peak (Fig. 6) and the 1729 cm^−1^ peak (Fig. 7).

#### 1.1.2 Azimuthal profiles

**Fig. 8:**
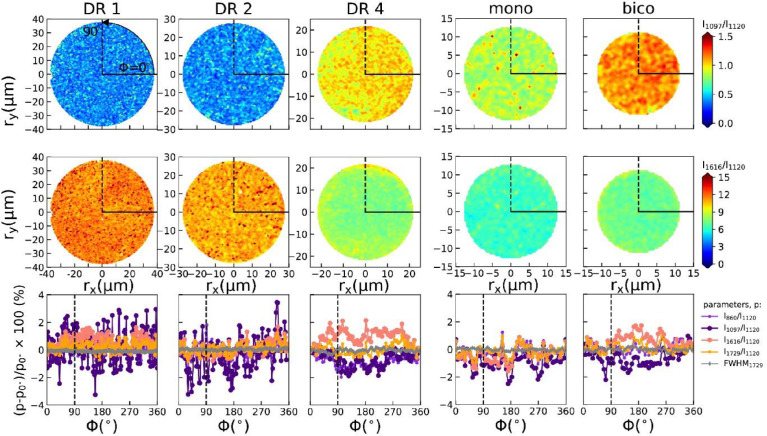
Top row: peak height ratio maps I_1097_/I_1120_ with annuli. Middle row: peak height ratio maps I_1616_/I_1120_. Bottom row: Azimuthal profiles of percentage changes in peak height ratios (I_860_/I_1120_, I_1097_/I_1120,_ I_1616_/I_1120_, I_1729_/I_1120_) and FWHM of 1729 cm^−1^ peak with respect to ϕ=0°.

Azimuthal profiles were extracted by converting the maps into polar coordinates and averaging the parameters (peak height ratios or FWHM) over the values having the same azimuthal angle φ. The maps of the intensity ratios and corresponding azimuthal profiles are shown in Fig. 8.

#### 1.1.3 2D correlation maps

**Fig. 9:**
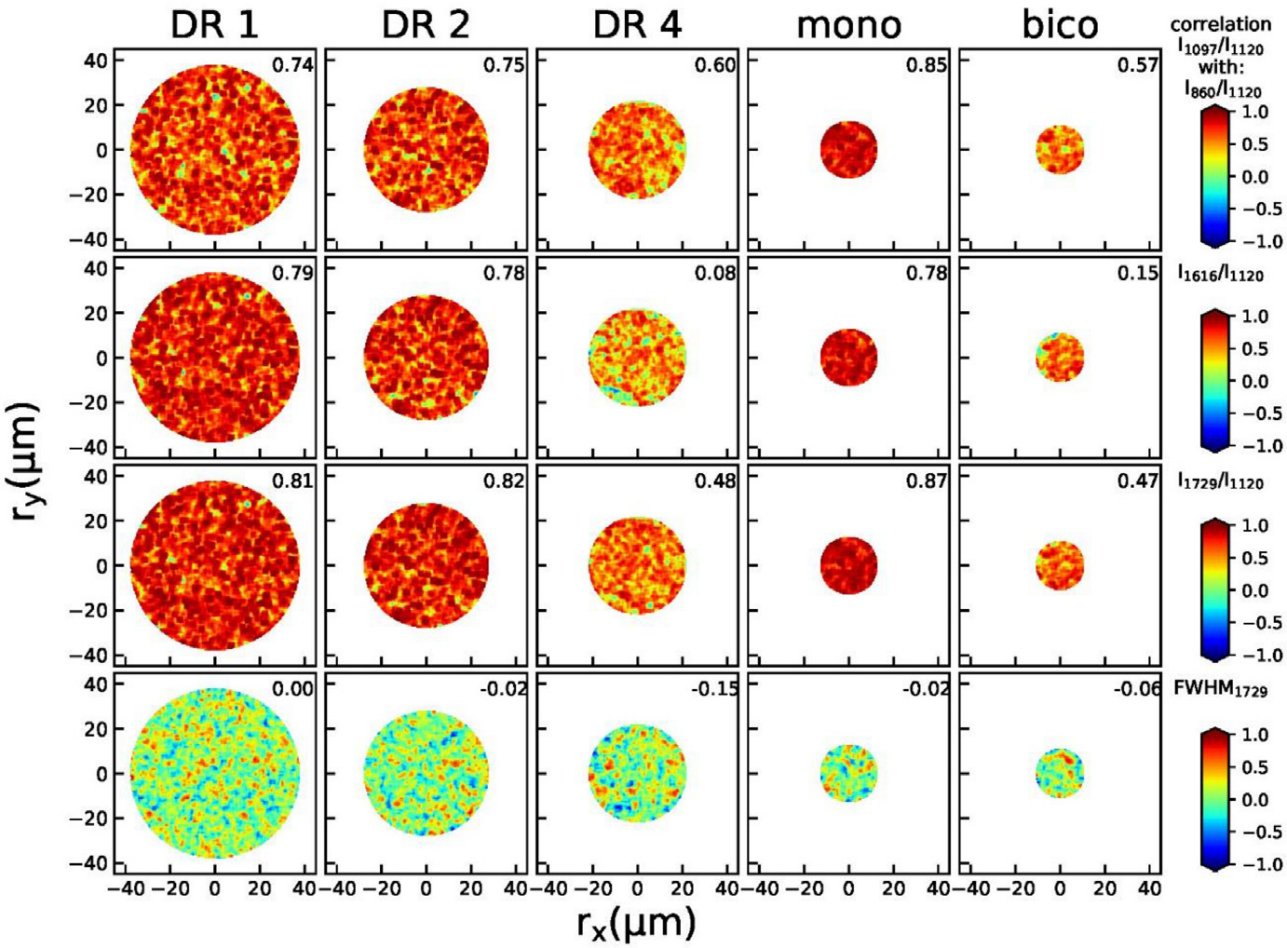
Calculated correlation maps for fibers DR1, DR2 and DR4 between peak height ratios (I_860_/I_1120_, I_1616_/I_1120_, I_1729_/I_1120_) and FWHM of the peak at 1729 cm^−1^ with respect to the peak height ratio I_1097_/I_1120_. The total Pearson correlation coefficients of the entire maps are given in the top right corner of each figure.

Correlation maps (Fig. 9) between peak height ratios (I_860_/I_1120_, I_1616_/I_1120_, I_1729_/I_1120_) and FWHM of the peak at 1729 cm^−1^ with respect to the peak height ratio I_1097_/I_1120_ were calculated by determining the Pearson correlation coefficients within 5 × 5 bins spanning an area of 2.5 × 2.5 µm. The correlation coefficients indicate that the peak height ratios for DR1 and DR2 are strongly positively correlated over the entire fiber cross-section, whereas the full width at half max of the peak at 1729 cm^−1^ is not correlated with the peak height ratio I_1097_/I_1120_ within the samples. For DR4, the peak height ratios are also positively correlated for I_860_/I_1120_ and I_1729_/I_1120_ with I_1097_/I_1120._ The peak ratio I_1616_/I_1120_ is not as strongly correlating with the peak ratio I_1097_/I_1120_.

### 1.2 ATR-FTIR data

**Fig. 10:**
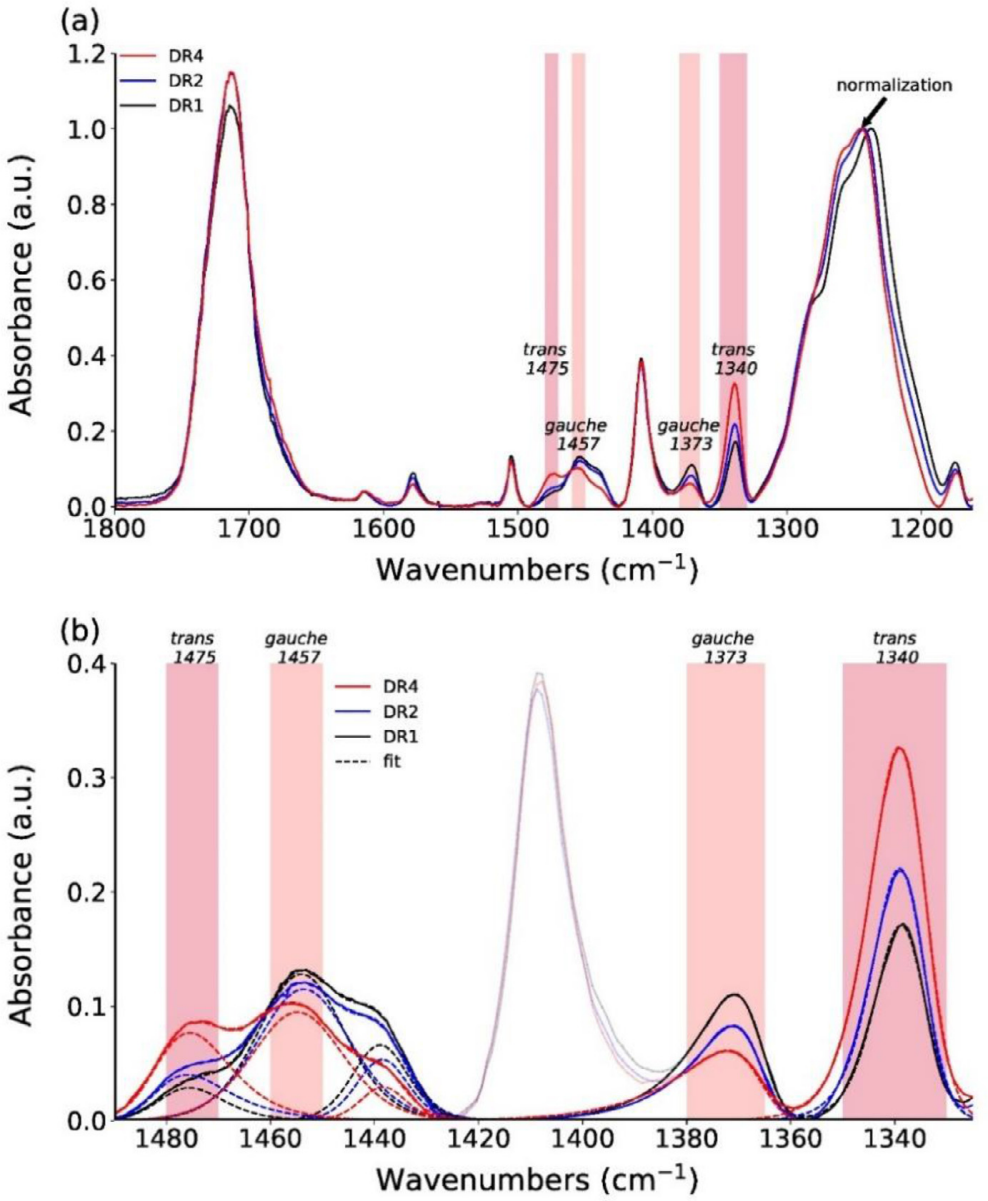
(a) Normalized ATR-FTIR spectra of DR1, DR2 and DR4 samples. The peak at 1245 cm^−1^ is used for normalization. (b) A close-up of normalized measured FTIR spectra (full lines), including fits (dashed lines).

We have measured ATR-FTIR spectra for fibers DR1, DR2 and DR4, in order to compare the extracted surface crystallinity values to the ones obtained from the Raman mapping of the outmost annulus (see article by E. Perret et al. [1]). ATR-FTIR spectra can be found online in the Mendeley repository [15]. Examples of background-corrected and normalized ATR-FTIR spectra are shown in Fig. 10a. The spectra were normalized by dividing the measured absorbance by the maximum absorbance in the range from 1225 to 1275 cm^−1^. This peak arises from ring and O—C stretching and corresponds to the Raman peak at a Raman shift of 1291 cm^−1^, which has also been used to normalize Raman spectra. In analogy to Raman studies, FTIR studies on PET have also reported that IR bands near 1475 and 1340 cm^−1^ increase in intensity during crystallization due to *trans* conformations of the ethylene glycol segment in crystalline PET [2]. Bands having wavenumbers close to 1457 and 1373 cm^−1^ have been associated with the *gauche* conformation of PET, and are typically reducing in intensity during crystallization. These *gauche* conformations are only present in the amorphous regions of PET, while *trans* conformations are present in both, crystalline and amorphous regions. Table 1 summarizes the assignments of these characteristic infrared bands to vibrations in PET.

Shaded regions in Fig. 10a highlight these characteristic infrared bands, which have been used to evaluate differences in surface crystallinities between individual samples, by analyzing the individual peak areas via curve fitting. Fig. 10b shows a close-up of the measured data in the 1325 to 1490 cm^−1^ region, including the fits. The band at 1410 cm^−1^ was excluded from the fits and is therefore greyed out in Fig. 10b. Upon drawing, peak heights of *gauche* ethylene glycol conformations are reducing, while those of *trans* are increasing.

**Fig. 11:**
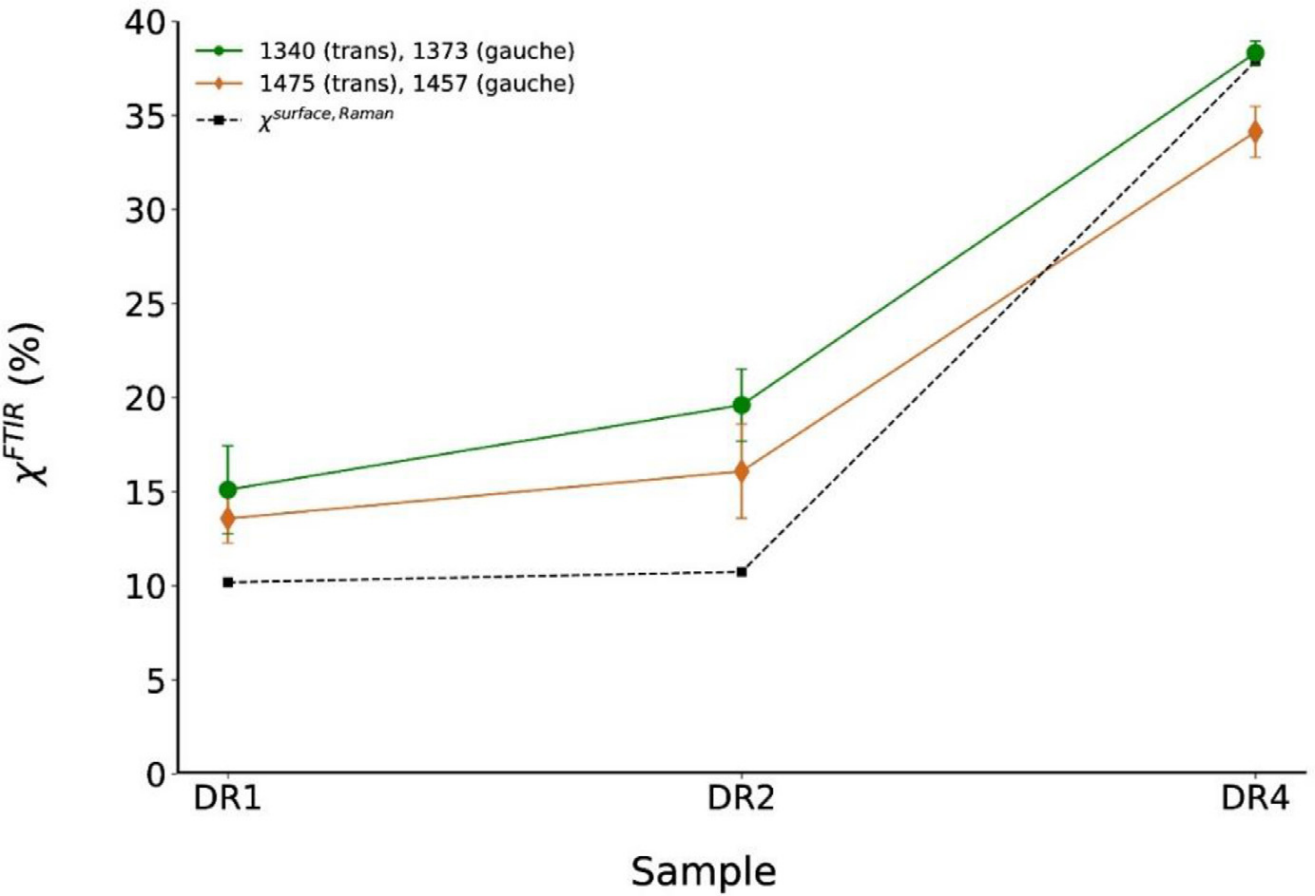
Surface crystallinity determined with ATR-FTIR. Error bars correspond to standard deviations in the determined crystallinities from three data sets. The dotted curve shows the Raman surface crystallinities of the out-most annuli of measured fiber cross-sections.

The calculated surface crystallinities from ATR-FTIR peak areas are shown in Fig. 11 and are compared to the surface Raman crystallinities of the outmost analyzed annulus in the fiber cross-sections. Extracted crystallinity values from ATR-FTIR spectra are remarkably close to extracted surface crystallinity values from Raman.

### 1.3 WAXD/SAXS data

As a complementary method, WAXD can be used to extract average crystallinity values of the fibers, and SAXS gives information about the arrangement of crystals and crystallite sizes. The WAXD and SAXS patterns are given online in the Mendeley repository [15].

**Fig. 12:**
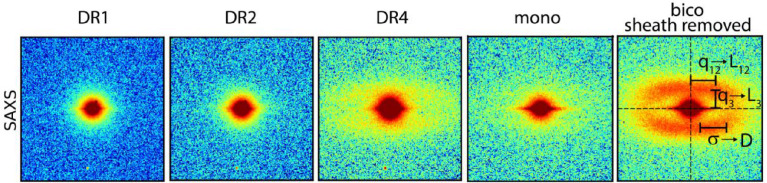
SAXS images for all fibers. Note that for the bicomponent fiber, the sheath was removed.

**Fig. 13:**
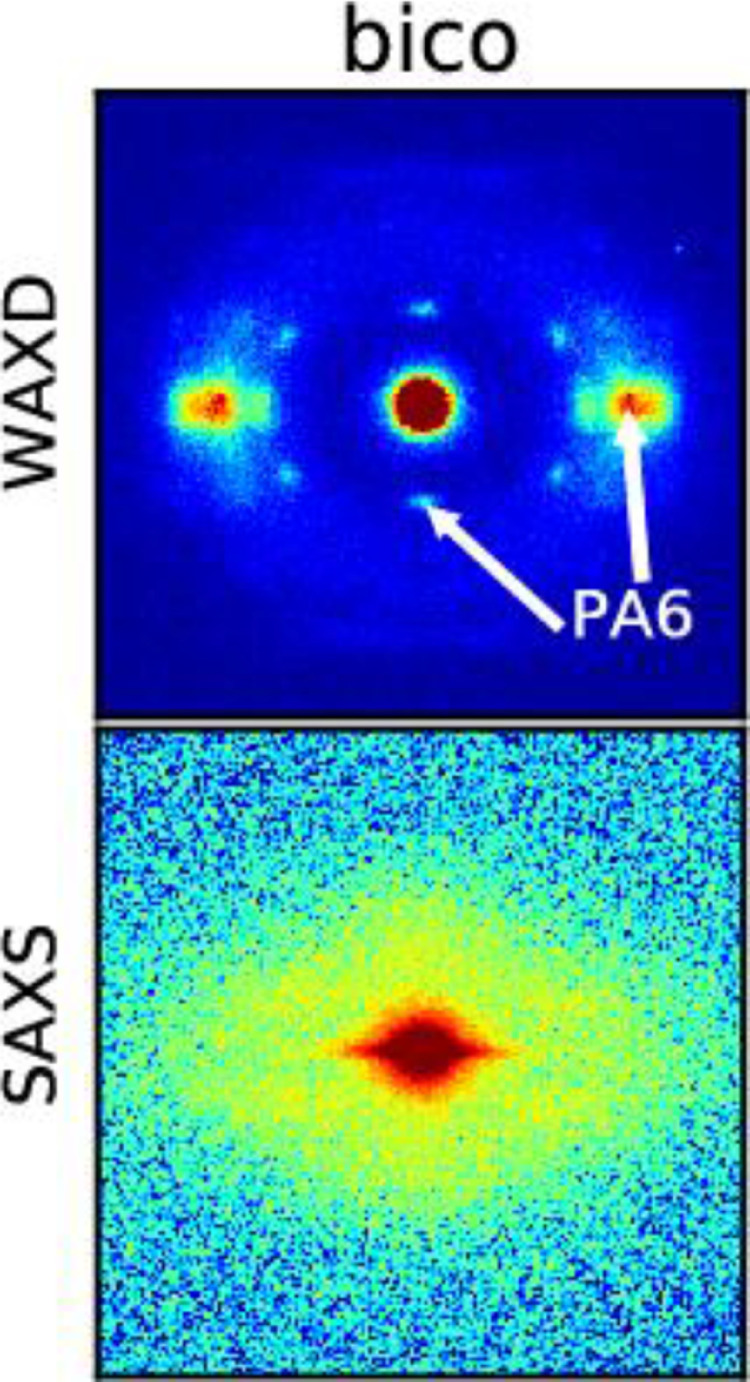
WAXD and SAXS patterns of bicomponent (bico) fiber.

Measured SAXS patterns of fiber samples DR1, DR2, DR4, mono and sheath-removed bico are shown in Fig. 12. Note that for the bicomponent fiber (bico), the sheath was removed following the procedure that is explained in the article by Perret et al. [1]. Patterns of the bico fiber including the sheath are shown in Fig. 13. The WAXD patterns are already discussed in detail in the article by Perret et al. [1]. Crystallinity values can be estimated from the WAXD patterns by analyzing the percentage of crystalline intensities in azimuthal or radial profiles (see experimental section). The determined crystallinity values are summarized in the article by Perret et al. [1] and are compared to the values obtained from other analytical methods like DSC.

In the SAXS patterns, the fibers DR4, mono and bico, as well as the core of the bico fiber, show lamellar four-point reflections of PET. Note that the lamellar four-point reflections of the mono fiber are weaker than the ones of DR4 and bico fiber.

Table 2 summarizes the long-spacings and crystal widths for the samples DR4, mono and sheath-removed bico, which have been determined from meridional and transversal profiles of the lamellar reflections. Details about the structural analysis from SAXS patterns are given in the experimental section. The mono fiber has slightly larger lateral crystallite sizes, *D*, than the DR4 fiber, with equal long-spacings, *L*_3_, along the fiber axis, but slightly larger lateral spacings, L_12_. The bicomponent fiber shows smaller long-spacings, *L*_3_.

WAXD and SAXS patterns of the bicomponent fibers (sheath not removed) are shown in Fig. 13.

### 1.4 DSC data

**Fig. 14:**
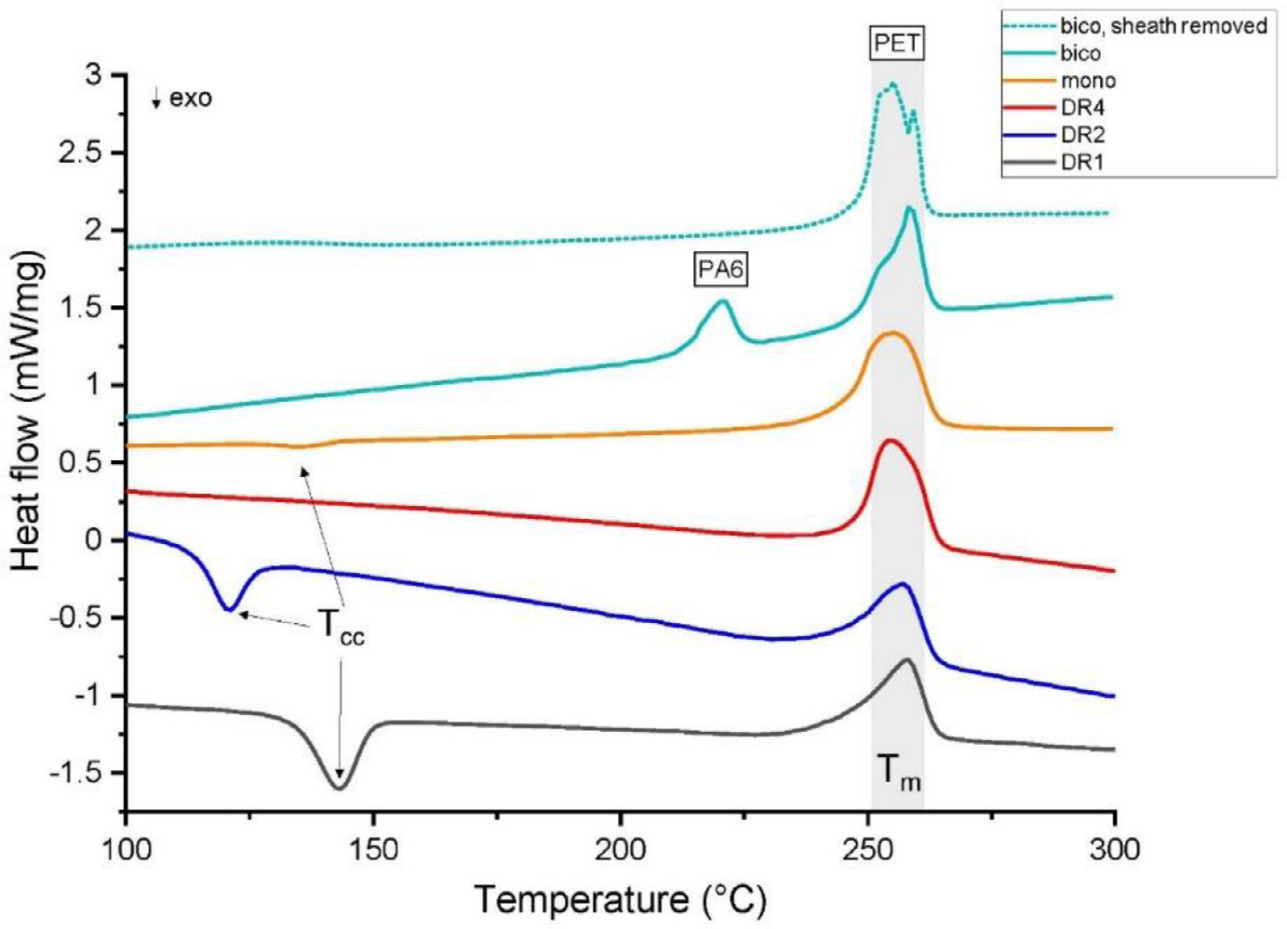
The DSC thermograms of first heating cycles of DR1 (lowest curve), DR2, DR4, mono and bico fibers, as well as bico fibers with removed sheath (top curve). Curves are offset for better visibility.

The average crystallinity and thermal properties of the fibers were analyzed using DSC. The DSC data files are given online in the Mendeley repository [15]. Fig. 14 shows the thermograms of the first heating cycles, which reflect the structural properties of all fibers.

**Table 1:** Assignments of analyzed infrared bands to vibrations in PET [2].

Peak position	Assignment
1340 cm^−1^ (*trans*), 1373 cm^−1^ (*gauche*)	glycol CH_2_ wagging
1475 cm^−1^ (*trans*), 1457 cm^−1^ (*gauche*)	glycol CH_2_ bending

**Table 2:** Structural parameters of the crystalline phase of PET, extracted from SAXS patterns.

Sample	*L_3_* (nm)	*L_12_* (nm)	*D* (nm)
DR4	14.9 ± 1.0	6.3 ± 0.4	5.8
mono	14.9 ± 1.0	7.4 ± 0.5	7.1
bico, sheath removed	12.9 ± 0.8	7.6 ± 0.5	6.6

Errors in the long-spacings were calculated using an uncertainty of 0.3° in the 2θ angle.

Cold crystallization temperatures (T_cc_), melting temperatures (T_m_), and corresponding enthalpies (ΔH_cc_, ΔH_m_), have been extracted from the data shown in Fig. 14, and the values are tabulated in Table 3. The crystallinity was calculated from the enthalpies (see experimental section).

A cold crystallization peak is observed for the as-spun fiber (DR1) and for the drawn fiber DR2, but is absent for the highly drawn fiber (DR4).

For the bicomponent fiber, a double melting peak for PET is observed. Such double peaks or peaks with shoulders may arise due to a melting/recrystallization of crystals, or due to crystals having different defect levels, which may also be a result of the preliminary cold-crystallization that happens in some of the samples.

The calculated percent crystallinity from the DSC curve of the as-spun fiber (DR1) shows that this fiber is practically amorphous (χ ∼8%). The increase in crystallinity from DR1 to DR4 can be seen by the decrease in the enthalpy of cold crystallization and by the increasing sharpness of the melting peak. The mono fiber shows a similar crystallinity value (χ ∼37.4%) as DR4 (χ ∼36.8%).

**Table 3:** Thermal properties and calculated crystallinity values of fibers from DSC.

Fiber label	T_cc_ (°C)	ΔH_cc_ (J/g)	T_m_ (°C)	$\Delta H_{m}^{PET}$ (J/g)	χ (%)
DR1	143.2	29.3 ± 2.1	257.9	40.4 ± 3.6	7.9 ± 1.2
DR2	121.0	25.3 ± 2.0	257.6	41.2 ± 3.5	11.4 ± 1.6
DR4	–	–	254.7	51.5 ± 0.8	36.8 ± 0.6
mono	140.9	1.8 ± 0.5	254.6	54.0 ± 0.7	37.4 ± 0.5
bico					
(PET component)	–	–	258.4	56.5 ± 2.4	40.4 ± 1.7
bico sheath removed					
(PET component)	–	–	259.8	58.4 ± 1.1	41.7 ± 1.1

The calculated crystallinity value of PET (χ ∼40.4%), from the measurement of the bico fiber, is very close to the one extracted from the melting of the same fiber, where the sheath has been removed before the measurement (χ ∼41.7%).

### 1.5 AFM data

**Fig. 15:**
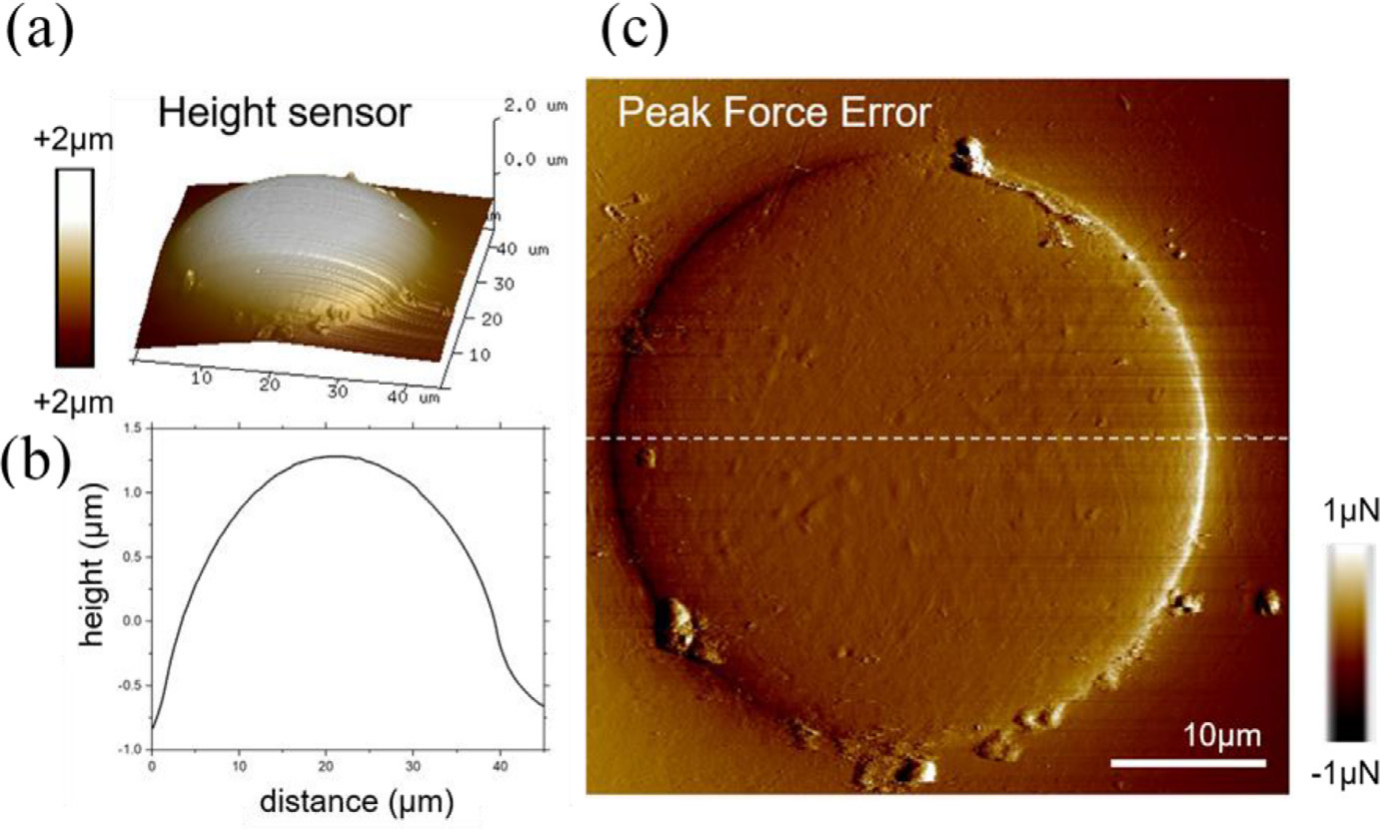
(a) 3D view of height sensor AFM image of embedded PET fiber. (b) Height profile across the face of the fiber. (c) Peak force error image.

Fig. 15 shows an AFM image with the peak force error and a height profile across the face of an embedded fiber. The fiber face is bent by a few micrometers. The AFM data file is given online in the Mendeley repository [15] and can be opened with the shareware NanoScope Analysis software (Version 1.9, Bruker AXS, Karlsruhe, Germany).

## 2 Experimental Design, Materials and Methods

The materials and experimental methods for Raman mapping and WAXD measurements have been previously described in detail in the article by Perret et al. [1]. Text below, which has been put into quotes, has been taken from said article.

### 2.1 Materials

'Three PET monofilaments were studied, which are labeled DR1, DR2 and DR4. A melt-spun amorphous PET fiber (DR1), which was directly wound up after melt-spinning without drawing, was provided by Monosuisse AG (Emmenbrücke, Switzerland). This filament was melt-spun from PET polymer pellets from Invista (Wichita, Kansas, United States), which had an intrinsic viscosity of about 0.63 dl/g. This as-spun fiber was used as provided to produce two drawn fibers (DR2 and DR4) with draw ratios 2 and 4, respectively. The draw ratios represent the ratios between the winder speed and first godet speed. The offline drawing was performed at Empa (St. Gallen, Switzerland) with a custom-made drawing setup using three godets and a heating plate. (..)

In addition, bicomponent PET-PA6 (core-sheath) filaments, as well as a monocomponent PET multifilaments were produced via direct spinning using a custom-made pilot plant at Empa (St. Gallen, Switzerland). (..)

The studied single filaments out of multifilaments are named (bico) for the bicomponent PET(core)-PA6(sheath) fiber and (mono) for the melt-spun PET monocomponent filament. (..) '

For further information regarding processing parameters, draw ratios, fineness and filament diameters we refer the reader to the article by Perret et al. [1].

### 2.2 Offline drawing setup of PET filaments

**Fig. 16:**
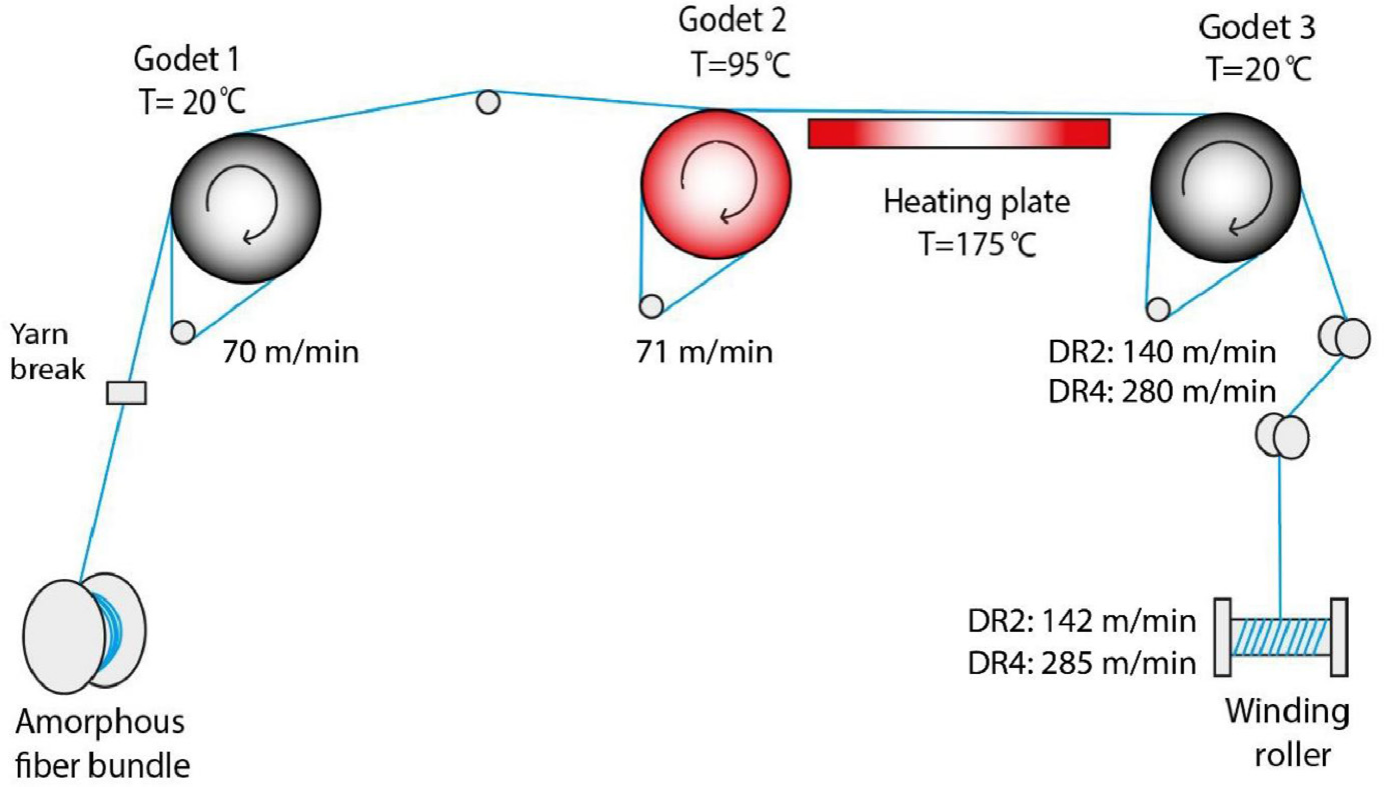
Illustration of the drawing setup at Empa, St.Gallen, Switzerland.

The drawing setup of PET filaments at Empa (St. Gallen, Switzerland) is illustrated in Fig. 16. The as-spun fiber, DR1, was drawn with this setup to thinner filaments with draw ratios DR = 2 and DR = 4.

### 2.3 High-resolution Raman mapping

**Fig. 17:**
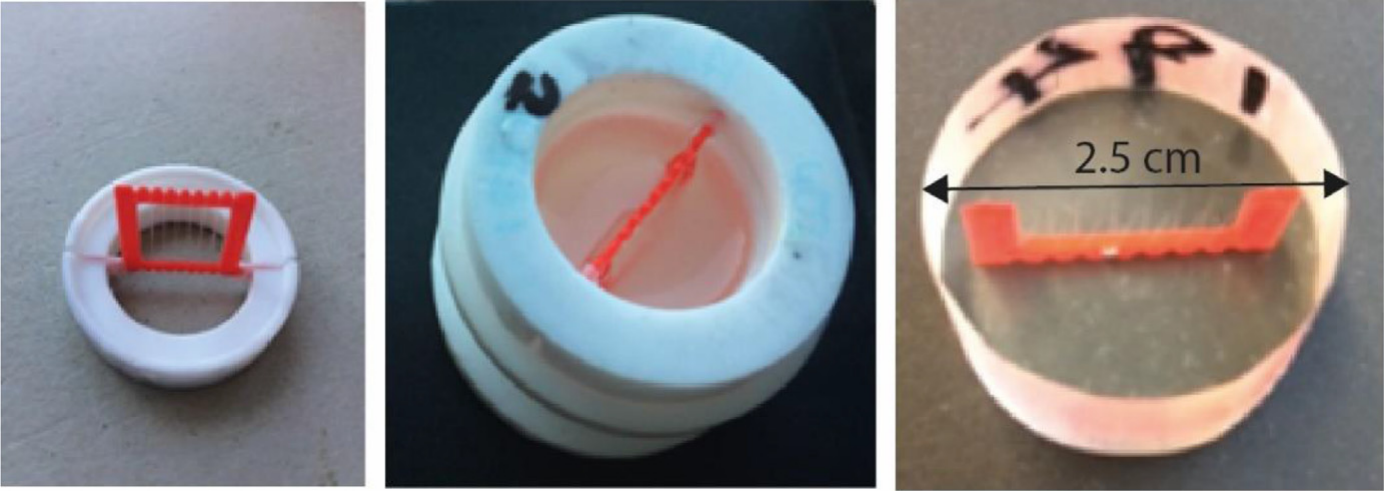
Left and middle: Sample holder and mold for fiber embedding. Right: embedded fibers.

Before the Raman measurement, the fibers were embedded in a resin hardener (Epoxi cure 2, Buehler, USA). First, the fibers were wound around a sample holder, which was subsequently inserted in the respective mold for embedding (Fig. 17). A resin hardener (Epoxi cure 2, Buehler, USA) was poured into the mold under vacuum and the mold was left to cure overnight. After embedding, the sample cylinders were ground and polished several times to obtain a smooth and transparent surface for Raman spectroscopy measurements.

**Fig. 18:**
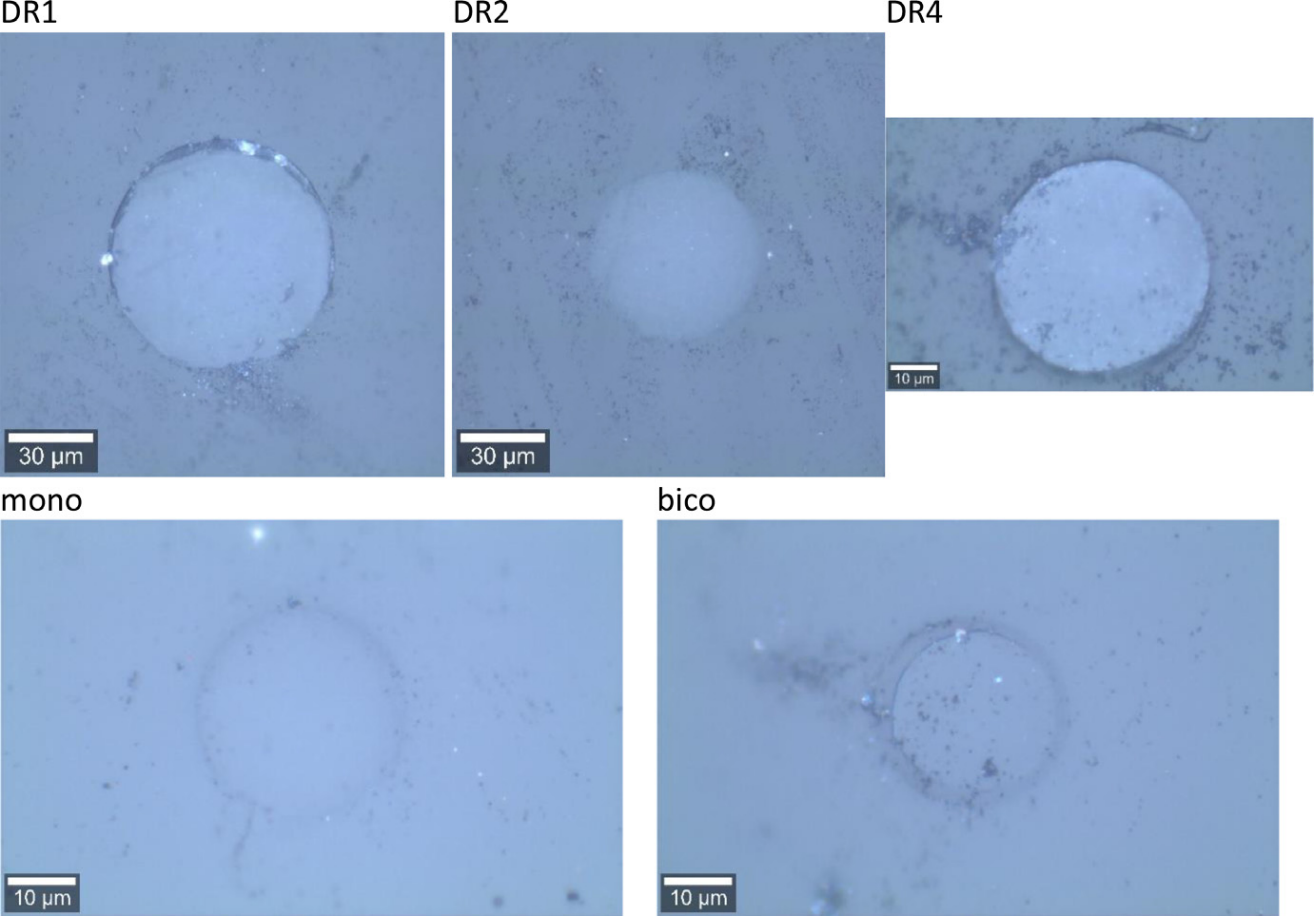
Microscope images of embedded fibers DR1, DR2, DR4, mono and bico.

Microscope images taken with the Raman setup are shown in Fig. 18.

'Raman spectra were acquired using a WITec Alpha 300 R confocal Raman microscope (WITec GmbH, Ulm, Germany) in backscattering geometry at Empa (Dübendorf, Switzerland). As an excitation source, a blue laser with 488 nm wavelength was used. The light was focused onto the embedded sample using a 100 × objective with a numerical aperture of 0.9, resulting in a diffraction-limited in-plane laser spot size of < 1 µm. The confocality of the Raman microscope limits the focal depth to approximately < 1 µm. The Rayleigh scattered light was blocked by a notch filter. The backscattered light was coupled to a 300 mm lens-based spectrometer with a grating of 1800 g/mm for all filaments. The spectrometer is equipped with a thermoelectrically cooled CCD (1600 × 200 pixel, pixel size 16 × 16 μm^2^) leading to a spectral resolution of < 0.85 cm^−1^. Raman spectra were acquired with a set laser power of 1 mW and an integration time of 2.0 s for the mono and bico filaments, and a laser power of 5 mW with an integration time of 0.4 s for DR1, DR2 and DR4, respectively.

The embedded samples were mounted on a piezo stage and maps of fiber cross-sections were acquired by scanning the sample through the laser. The raw spectra have been treated with a cosmic ray removal procedure and a background was subtracted using a moving shape with a radius of 100 cm^−1^ in order to remove signatures of photoluminescence. All corrected Raman spectra were subsequently binned by averaging over 4 spectra (2 × 2), spanning an area of 1 µm^2^. (…)'

For further analysis details, like fitting procedures, we refer the reader to the article by Perret et al. [1].

### 2.4 ATR-FTIR spectroscopy

**Fig. 19:**
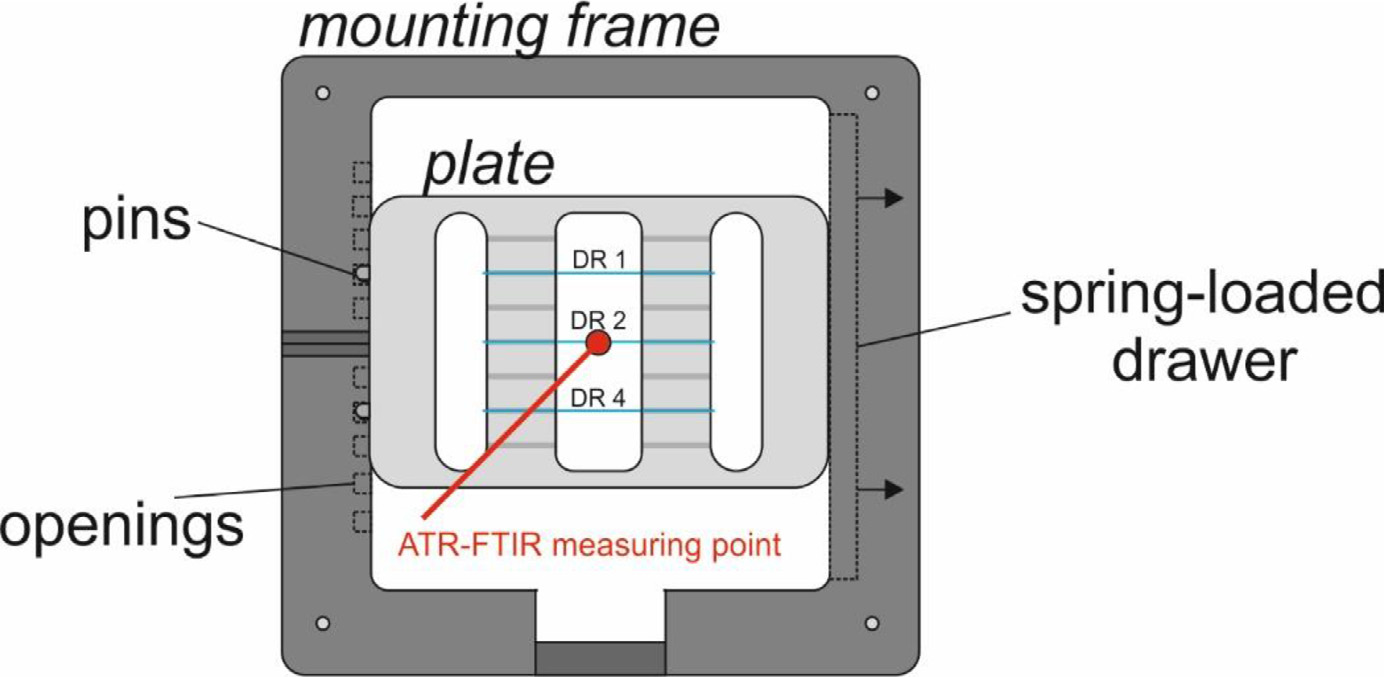
Mounting frame and sample holder plate for ATR-FTIR measurements.

The as-spun DR1 and drawn PET monofilaments DR2 and DR4 were analyzed with ATR-FTIR. ATR-FTIR spectra were recorded with a Bruker Tensor 27 FTIR spectrometer (Bruker Optics, Ettlingen, Germany), using a single reflection attenuated total reflectance (GladiATR™) accessory from Pike Technologies (Fitchburg, Wisconsin, United States). The ATR accessory is equipped with a monolithic diamond ATR crystal. In order to repeatedly achieve the same position and orientation of the fibers on the ATR accessory, we have custom-made a sample holder system. The system consists of a mounting frame, which is fixed into place on the table top of the GladiATR. A sample holder plate is mounted into the frame by using the spring-loaded drawer on the right-hand side of the frame, and two pins are attached to the left side of the plate, which snap into specific openings of the frame (Fig. 19). This sample holder plate has very fine grooves for fiber mounting. Fibers are positioned in the grooves and are fixed to the plate with adhesive tape. Between each measurement, the fibers can easily be switched by snapping the sample holder into the respective frame openings. A background was acquired immediately before each fiber measurement by measuring an empty slot on the sample holder.

The FTIR spectrometer uses a mid-infrared (MIR) Globar source and a narrow-band mercury cadmium telluride (MCT) detector. The infrared light is guided with several optical parts to the sampling surface with an angle of incidence of 45° The depth of penetration of the infrared light into the PET sample can be calculated using the following equation [3]:

(1) $$\Delta z=\frac{\lambda }{2\pi n_{1}{\left(sin^{2}\theta -{\left(n_{2}/n_{1}\right)}^{2}\right)}^{0.5}}$$

where λ is the wavelength of interest, $n_{1}=2.40$ and $n_{2}\approx 1.545$ are the refractive indices of the ATR crystal and the PET polymer (dispersion is neglected), respectively. For wavenumbers ranging from 1470 to 840 cm^−1^ (λ = 0.00068 to 0.00119 cm), the calculated penetration depth, *Δz*, varies from 1.5 to 2.7 µm.

Reproducible contact pressure between the ATR crystal and the sample was ensured by handling the pressure clamp of the ATR system in the exact same way for each measurement. Absorbance spectra spanning wavenumbers between 4000 and 600 cm^−1^ were collected with a spectral resolution of 2 cm^−1^. Each fiber was measured three times. For each spectrum, a total of 32 scans has been taken and averaged. To minimize differences between spectra due to baseline shifts, the spectra were cut in the range of 4000 to 700 cm^−1^, due to high noise below 700 cm^−1^, and the baselines were subsequently subtracted by using a concave Rubber band algorithm with 20 iterations using OPUS™ software (Version 8.5, Bruker AXS, Karlsruhe, Germany). Additional data analysis such as normalization and peak fitting have been performed with specifically developed Python codes. The ATR-FTIR files given in the Mendeley repository [15] can be opened with Python codes using the open source opusFC Python module (https://pypi.org/project/opusFC/).

#### 2.4.1 ATR-FTIR analysis: surface crystallinity

The surface crystallinities of samples DR1, DR2 and DR4 have been estimated from the peak areas, A_1340_, A_1373_, A_1475_, A_1457_, of the two *trans* and *gauche* pairs (Table 1) in the ATR-FTIR spectra of the fiber samples and from the peak areas, A_1340a_, A_1475a_ from an amorphous sample, using the following equations:

(2) $$\chi^{FTIR}\left(\%\right)=\frac{A_{crys.\left(trans\right)}}{A_{\left(trans\right)}+\gamma A_{\left(gauche\right)}}\times \;100$$

(3) $$\chi_{1340}^{FTIR}\left(\%\right)=\frac{A_{1340}-A_{1340a}}{A_{1340}+\gamma_{1373}A_{1373}}\times \;100$$

(4) $$\chi_{1475}^{FTIR}\left(\%\right)=\frac{A_{1475}-A_{1475a}}{A_{1475}+\gamma_{1457}A_{1457}}\times \;100$$

Note that it is necessary to take into account the difference in the absorptivity, γ, of the *trans* and *gauche* conformations [4,5].

Specific peaks arising from *trans* and *gauche* conformations of the ethylene glycol segment were fit with asymmetric Pearson VII functions, where the peak width is allowed to vary sigmoidally [6]. The function of the asymmetric Pearson VII function is given by the following equation:

$$I_{sim}^{FTIR}=I\frac{FWH{M_{asym}}^{2m}}{{\left[FWH{M_{asym}}^{2}+\left(2^{1/m}-1\right){\left(x-x_{0}\right)}^{2}\right]}^{m}}$$

(5) $$FWHM_{asym}=\frac{2\text{FWHM}}{1+e^{-a\left(x-x_{0}\right)}}$$

with *a* being the asymmetry factor, *FWHM_asym_* the asymmetric peak width, *x*_0_ the peak position, *I* the peak intensity and *m* the shape factor.

**Fig. 20:**
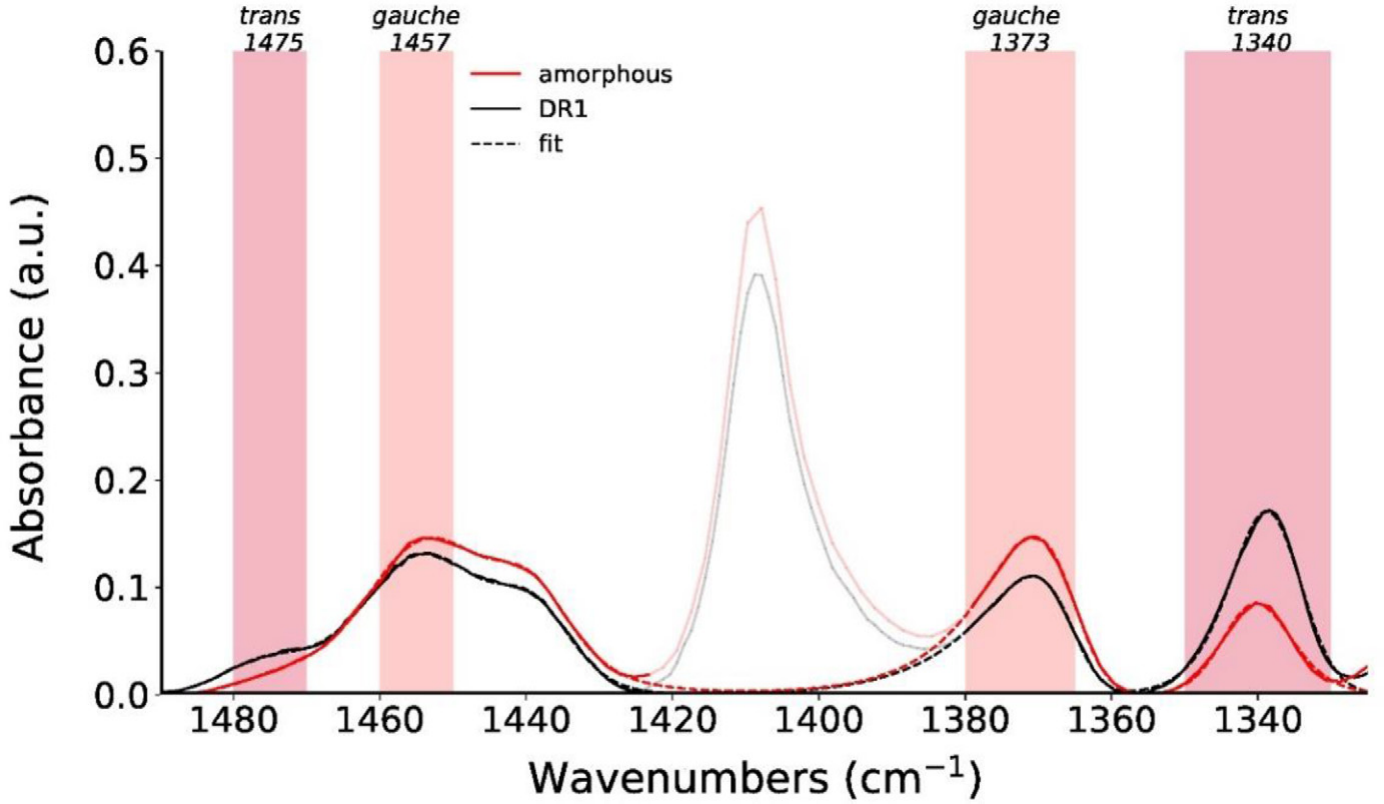
ATR-FTIR spectra of amorphous PET film and DR1. Fits are shown as dashed lines.

The infrared band at 1340 cm^−1^ (*trans*) is also present in fully amorphous PET samples, and its peak area has been estimated to be about $A_{1340a}=1.0$ from the normalized spectrum of an amorphous PET film (Fig. 20). The band at 1410 cm^−1^ was excluded from the fits and is therefore greyed out in Fig. 20. Fits are shown as dashed lines. Note that the fits reproduce extremely well the measured data and they can therefore hardly be distinguished from the raw data. The infrared band at 1475 cm^−1^ is negligibly small in amorphous samples ($A_{1475a}=0$).

**Fig. 21:**
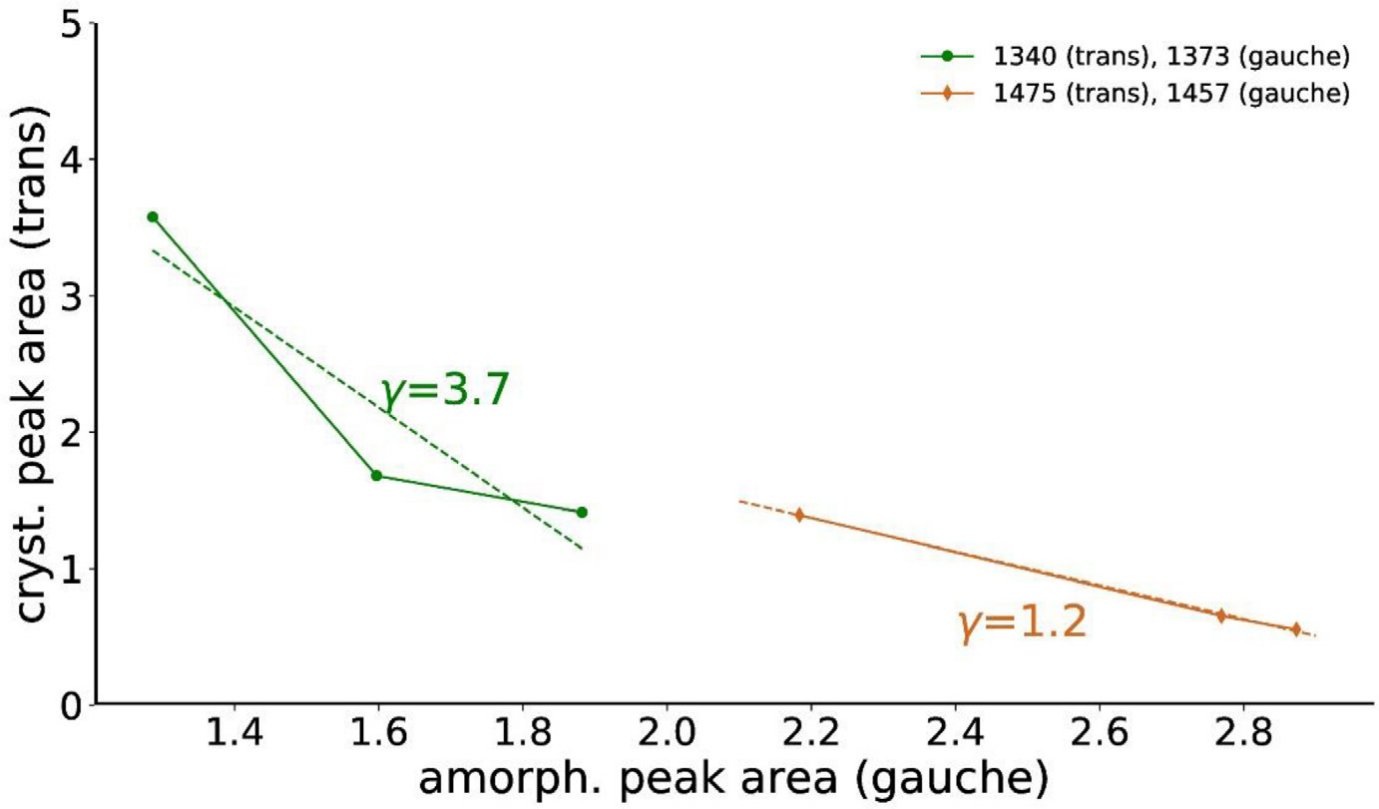
The crystalline peak area from peaks with *trans* conformation are plotted against the amorphous peak area from *gauche* peaks. The slope is fit (dashed curves) to extract the absorptivities.

The absorptivities were determined by plotting the *trans* versus *gauche* integrated peak areas. The individual peak absorptivities ($\gamma_{1373}=3.7,\;\gamma_{1457}=1.2$) were calculated from the slopes of the linear fits (Fig. 21).

### 2.5 WAXD and SAXS

WAXD and SAXS patterns were recorded on a Bruker Nanostar U diffractometer (Bruker AXS, Karlsruhe, Germany) with a Cu-Kα radiation λ = 1.5419 Å and a VÅNTEC-2000 MikroGap area detector. Single filaments (DR1, DR2, DR4) or fiber bundles (mono, bico, bico with sheath removed) were mounted on sample holders for WAXD, and the sample to active detector area distance was about 9.3 cm.'

For SAXS measurements, fiber bundles were mounted for all fiber types, and the sample to active detector area distance was about 111 cm.

'The recorded patterns were analyzed with the evaluation software DIFFRAC.EVA (version 4.2., Bruker AXS, Karlsruhe, Germany) and specifically developed Python codes. For the samples, DR4, mono and bico with sheath removed, the crystallinity values of PET (percentage of crystalline intensity in WAXD patterns) were estimated from Lorentz corrected 2D patterns by analyzing azimuthal and radial profiles using DIFFRAC.EVA. The Lorentz correction has been explained elsewhere' [7]. '(…)'

Long-spacings and transversal crystallite sizes were extracted by fitting meridional and transversal profiles and applying Bragg's law and the Scherrer equation, respectively (section 2.5.2). Note that the Bruker (.gfrm) images from the Mendeley repository [15] can all be plotted with the open source Fabio python package (https://pypi.org/project/fabio/).

#### 2.5.1 WAXD analysis: crystallinity estimations

**Fig. 22:**
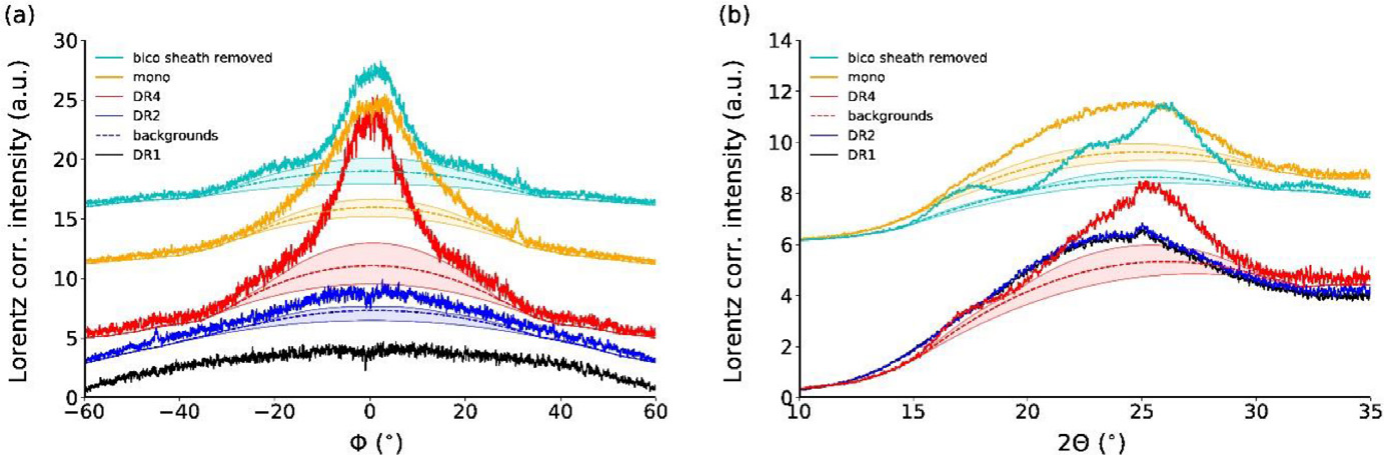
Lorentz corrected (a) azimuthal profiles (between 2θ = 12–32°, *φ* = 0° corresponds to the equator) and (b) radial profiles (offset for better visibility) of all samples. Estimated amorphous phase contributions using DIFFRAC.EVA software are shown as dashed lines. Areas between lower and upper bounds for the amorphous phase contribution are shaded.

Fig. 22a shows Lorentz corrected azimuthal profiles and Fig. 22b shows radial profiles extracted from WAXD patterns of fibers, DR1, DR2, DR4, mono and bico sheath removed. Estimated amorphous phase contributions using DIFFRAC.EVA software are shown as dashed lines.

The WAXD patterns of fibers DR2, DR4, mono and bico (sheath removed) have shown an overlap of several scattering features: amorphous phase, crystalline phase and a highly oriented non-crystalline mesophase (P_nc_) [8], [9], [10], [11]. While the amorphous phase is completely unoriented, the non-crystalline mesophase P_nc_ is composed of oriented, conformationally disordered macromolecules. The contribution of the amorphous/P_nc_ phase to the azimuthal profiles of DR2 and DR4, was estimated with the DIFFRAC.EVA (version 4.2., Bruker AXS, Karlsruhe, Germany) software using a curvature of *c* = 0.2 for the bico (sheath removed) fiber, *c* = 0.25 for DR4 and *c* = 0.35 for the mono fiber. Lower and upper bounds correspond to *c*-0.1 ≤ *c* ≤ *c* + 0.2.

In case of the azimuthal profiles, the intensity percentage calculation of the P_nc_ phase in DR2 and of crystallinity calculation in DR4, mono and bico (sheath removed) fibers is done as follows:

(6) $$\chi \text{WAXD}\left(\%\right)=\frac{I_{\text{total}}-I_{\text{amorph}/\text{Pnc}}}{I_{\text{total}}}\times 100$$

where *I*_total_ stand for the total integrated intensity of the azimuthal/radial profiles and *I*_amorph/Pnc_ for the estimated background (dashed lines in Fig. 22) arising from the amorphous phase (in DR2) and amorphous and P_nc_ phase in DR4, mono and bico sheath removed.

In case of the radial profile, the same equation can only be applied for the DR4, mono and bico sheath removed fibers. In DR2, the radial summation of the P_nc_ and amorphous phase leads to practically the same radial profile as DR1 and can therefore not be analyzed. The crystalline signal contribution to the WAXD pattern was estimated from the radial profile in the range between 10 and 35°. The amorphous phase contribution in radial profiles was estimated for DR4, bico sheath removed and mono fibers. The curvature was chosen to be *c* = 3.3 for the amorphous phase backgrounds of the radial profiles of DR4 and bico sheath removed and a curvature of *c* = 4.3 was chosen for the mono fiber. The lower and upper bounds correspond to *c*-1.0 ≤ *c* ≤ *c* + 2.0.

#### 2.5.2 SAXS analysis: long-spacings and crystallite sizes

**Fig. 23:**
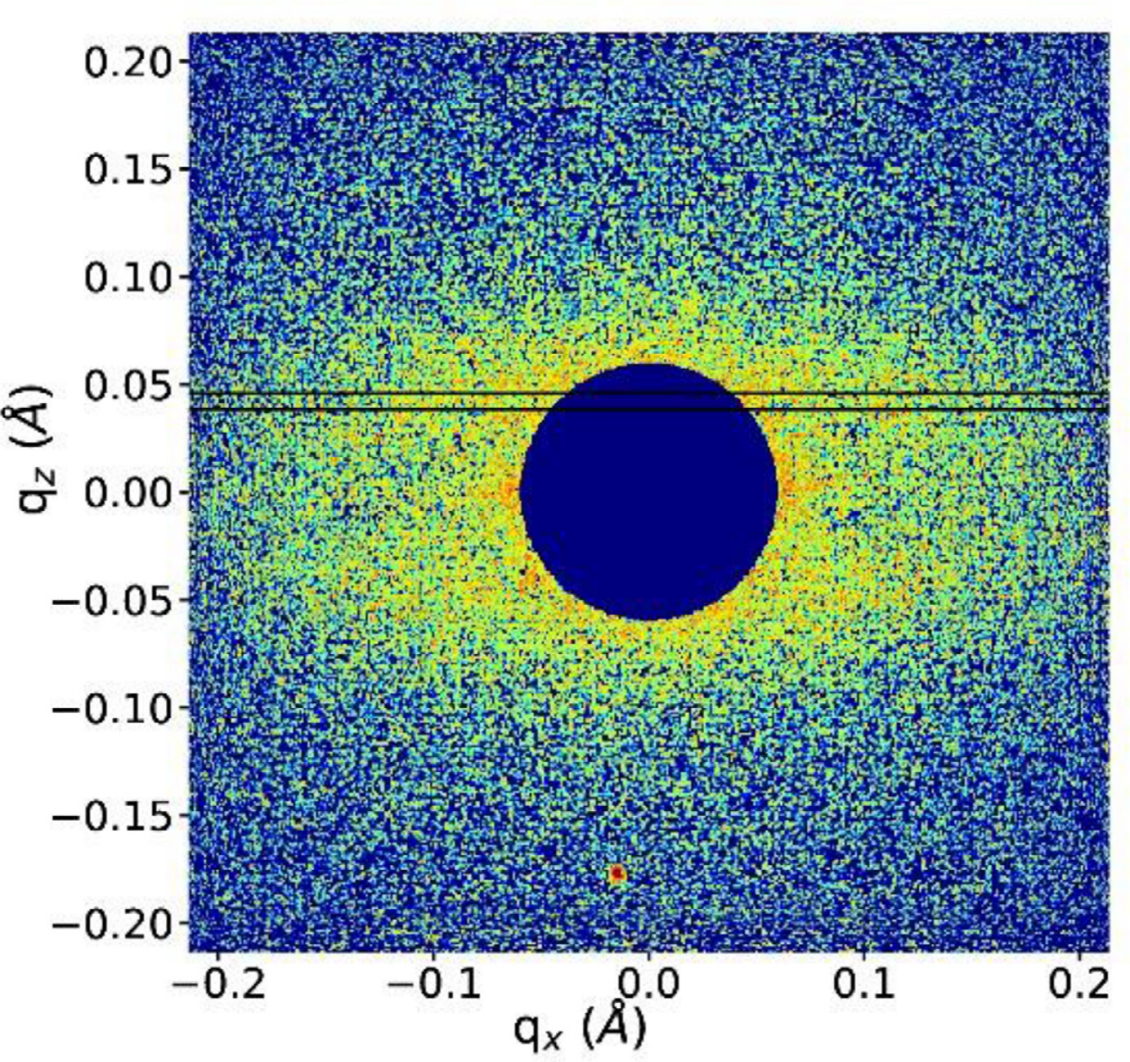
SAXS image of a bundle of DR4 fibers. The direct beam has been masked. The area between the horizontal lines (transversal area) was used to extract transversal profiles.

**Fig. 24:**
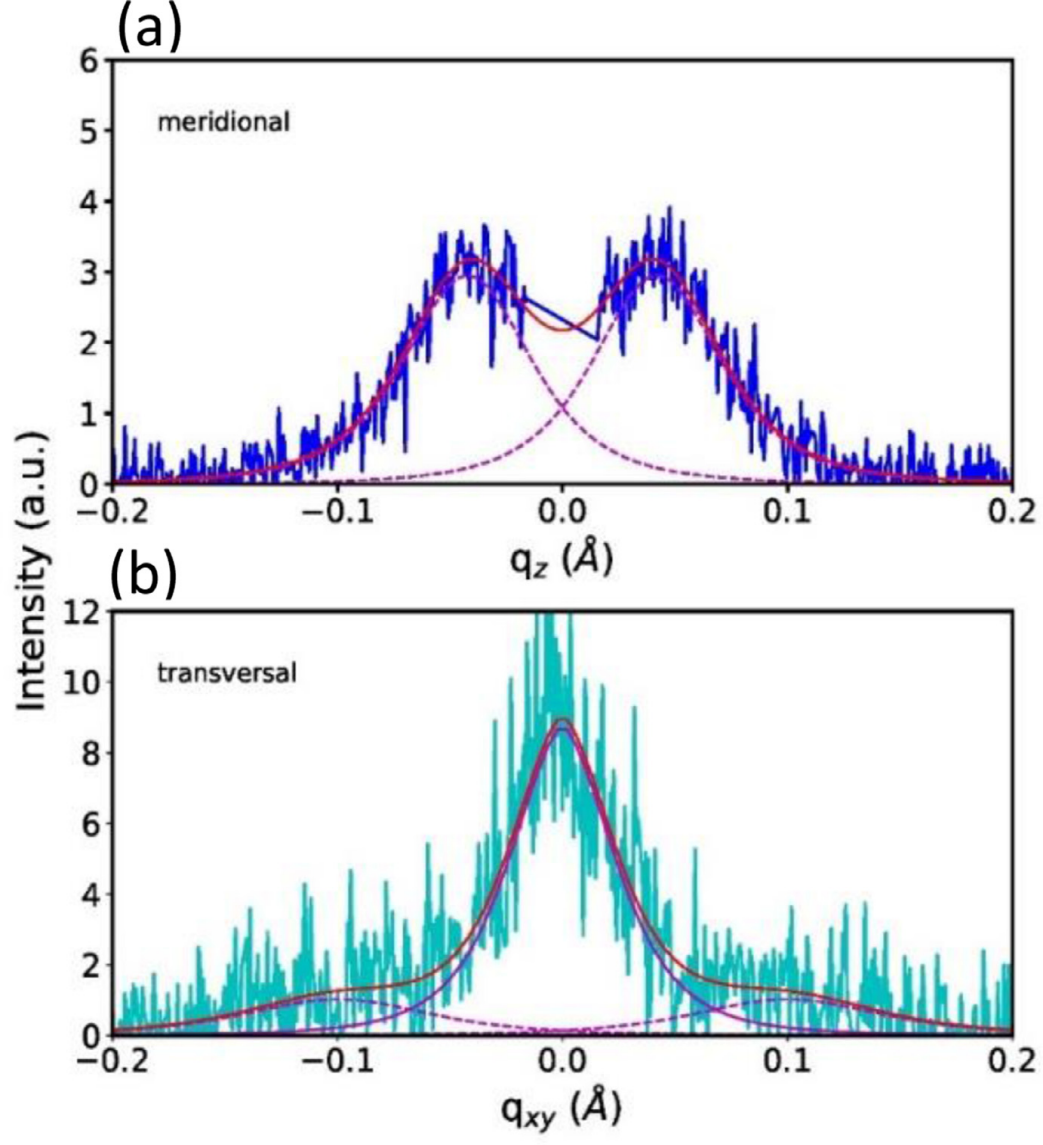
(a) meridional and (b) transversal profiles of DR4. Total fits are shown as red lines and dashed lines are the lamellar peak fits.

The area of the direct beam in the SAXS patterns was masked (Fig. 23) and the meridional profile was obtained by projecting the patterns onto the *q_z_* axis (vertical momentum transfer) [12]. The long-spacing *L*_3_ was then obtained by fitting the meridional profile with two pearson VII functions with shape factor 2 and applying the Bragg equation (Fig. 24a):

(7) $$L_{3}=\frac{2\pi }{q_{\text{LM}}}=\frac{\lambda }{\left(2\sin\theta_{\text{LM}}\right)}$$

where $q_{LM}=\frac{4\pi }{\lambda }\sin\theta_{LM}$ is the scattering vector, $\theta_{LM}\;$is half the scattering angle at the lamellar reflection and λ is the wavelength.

Transversal profiles (Fig. 24b) were obtained by projecting transversal areas (area between horizontal lines in Fig. 23) around the lamellar reflection onto the in-plane momentum transfer, *q*_xy_ axis. This profile was then fit with pearson VII functions of shape factor 2. The lateral long-spacing, *L*_12_, between fibrils was then calculated from the position of the lamellar reflections on the *q*_xy_ axis by applying Bragg's law and the crystallite sizes were calculated from the width of the lamellar reflections applying the Scherrer equation [13]:

(8) $$D=\frac{0.9\lambda }{\Delta \left(2\theta \right)\cos\theta }\approx \frac{0.9\lambda F}{\sqrt{\text{FWH}M^{2}-b^{2}}}$$

where *D* is the lamellar crystallite width, *F* is the sample to detector distance, *FWHM* is the full width at half-maximum of the reflection, and *b* is the instrumental broadening (b≈0).The equation makes use of small-angle approximations, cosθ≈1.

### 2.6 DSC

Average crystallinity values and thermal properties of all fibers were determined using DSC. Measurements were performed on the instrument DSC 214 Polyma (Netzsch, Selb, Germany) in a nitrogen atmosphere (40 mL/min). The fibers were cut into small pieces (∼ 2 mm long), and in each case about 5–10 mg of cut fibers were first heated from 25 to 300 °C, followed by a cooling step from 300 to 25 °C using a ramping rate of 10 °C/min. The data was analyzed using a DSC software (Netzsch Proteus Thermal Analysis, Version 7.1.0, Selb, Germany), to extract the crystallinity of individual samples.

#### 2.6.1 DSC analysis: crystallinity

The percent crystallinity of PET was calculated using the following equation:

(9) $$\chi \left(\%\right)=\frac{\Delta H_{m}-\Delta H_{cc}}{\Delta H_{m}^{0}}\times \;100$$

The value for heat of fusion of a 100% crystalline PET material ($\Delta H_{m}^{0}$) was taken to be 140 J/g [14].

For the bico fiber, the area under the PET melting peak was utilized to determine the percent crystallinity of the PET core. In this case, the melting enthalpy extracted from the DSC software had to be corrected in order to take into account the mass of the PET material in the DSC crucible. The mass of PET, *m_PET_*, was estimated from the core (*d_core_* = 23 µm) and fiber diameters (*d_fiber_* = 29 µm), as well as from the densities of PA6 (*ρ_PA6_* = 1.14 g/cm^3^) and PET (*ρ_PET_* = 1.4 g/cm^3^). The melting enthalpy for PET in the DSC measurement of the bicomponent fiber was thus calculated by applying the following equations:

(10) $$L=\frac{m_{tot}}{\rho_{PET}A_{PET}+\rho_{PA6}A_{PA6}}$$

(11) $$m_{PET}=\rho_{PET}A_{PET}L$$

(12) $$\Delta H_{m}^{PET}=\Delta H_{m}m_{tot}/m_{PET}$$

Where *L* and *m_tot_* are overall length and total mass of the fiber material in the DSC crucible, while *A_PET_* and A_PA6_ are the cross-section areas of PET core and PA6 sheath, respectively.

### 2.7 AFM

The topography of an embedded PET fiber was characterized using peak-force tapping mode atomic force microscopy AFM (Nanoscope, Bruker AXS, Karlsruhe Germany) with silicon probes from Bruker AXS (Model: Scanasyst-Air) with tip radius <12 nm (force constant

∼0.4 N m^–1^, resonance frequency in the range of 70 kHz). Height diagrams were recorded with a scan size of 45 µm and a scan speed of 0.5 Hz (512 × 512 points). The shareware Bruker NanoScope Analysis software (Version 1.9, Bruker AXS, Karlsruhe, Germany) was used for AFM analysis.

## CRediT authorship contribution statement

**K. Sharma:** Data curation, Methodology, Writing – review & editing. **O. Braun:** Validation, Investigation, Data curation, Writing – review & editing. **S. Tritsch:** Methodology, Writing – review & editing. **R. Muff:** Investigation, Data curation. **R. Hufenus:** Supervision, Project administration, Writing – review & editing. **E. Perret:** Software, Formal analysis, Investigation, Data curation, Writing – original draft, Visualization.

## Declaration of Competing Interest

The authors declare that they have no known competing financial interests or personal relationships which have, or could be perceived to have, influenced the work reported in this article.
